# Effect of TCF7L2 on the relationship between lifestyle factors and glycemic parameters: a systematic review

**DOI:** 10.1186/s12937-022-00813-w

**Published:** 2022-09-26

**Authors:** Somayeh Hosseinpour-Niazi, Parvin Mirmiran, Shabnam Hosseini, Farzad Hadaegh, Elaheh Ainy, Maryam S Daneshpour, Fereidoun Azizi

**Affiliations:** 1 Nutrition and Endocrine Research Center, Research Institute for Endocrine Sciences, Shahid Beheshti University of Medical Sciences, Tehran, Iran; 2 School of Human Nutrition, Faculty of Agricultural and Environmental Sciences, McGill University, Montreal, Quebec Canada; 3 Prevention of Metabolic Disorders Research Center, Research Institute for Endocrine Sciences, Shahid Beheshti University of Medical Sciences, Tehran, Iran; 4 Department of Vice Chancellor Research Affairs, Shahid Beheshti University of Medical Sciences, Tehran, Iran; 5 Cellular and Molecular Research Center, Research Institute for Endocrine Sciences, Shahid Beheshti University of Medical Sciences, Tehran, Iran; 6 Endocrine Research Center, Research Institute for Endocrine Sciences, Shahid Beheshti University of Medical Sciences, Tehran, Iran

**Keywords:** TCF7L2, Glycemic parameters, Dietary factors, Lifestyle factors

## Abstract

**Background:**

Among candidate genes related to type 2 diabetes (T2DM), one of the strongest genes is Transcription factor 7 like 2 (TCF7L2), regarding the Genome-Wide Association Studies. We aimed to conduct a systematic review of the literature on the modification effect of TCF7L2 on the relation between glycemic parameters and lifestyle factors.

**Methods:**

A systematic literature search was done for relevant publications using electronic databases, including PubMed, EMBASE, Scopus, and Web of Science, from January 1, 2000, to November 2, 2021.

**Results:**

Thirty-eight studies (16 observational studies, six meal test trials, and 16 randomized controlled trials (RCTs)) were included. Most observational studies had been conducted on participants with non-diabetes showing that TCF7L2 modified the association between diet (fatty acids and fiber) and insulin resistance. In addition, findings from meal test trials showed that, compared to non-risk-allele carriers, consumption of meals with different percentages of total dietary fat in healthy risk-allele carriers increased glucose concentrations and impaired insulin sensitivity. However, ten RCTs, with intervention periods of less than ten weeks and more than one year, showed that TCF7L2 did not modify glycemic parameters in response to a dietary intervention involving different macronutrients. However, two weight loss dietary RCTs with more than 1-year duration showed that serum glucose and insulin levels decreased and insulin resistance improved in non-risk allele subjects with overweight/obesity. Regarding artichoke extract supplementation (ALE), two RCTs observed that ALE supplementation significantly decreased insulin concentration and improved insulin resistance in the TT genotype of the rs7903146 variant of TCF7L2. In addition, four studies suggested that physical activity levels and smoking status modified the association between TCF7L2 and glycemic parameters. However, three studies observed no effect of TCF7L2 on glycemic parameters in participants with different levels of physical activity and smoking status.

**Conclusion:**

The modification effects of TCF7L2 on the relation between the lifestyle factors (diet, physical activity, and smoking status) and glycemic parameters were contradictory.

**PROSPERO registration number:**

CRD42020196327

**Supplementary Information:**

The online version contains supplementary material available at 10.1186/s12937-022-00813-w.

## Introduction

Type 2 diabetes (T2DM) has become a serious global health problem. The International Diabetes Federation has reported that 463 million adults were living with diabetes worldwide in 2019. This number is estimated to rise to 700 million by 2045 [1]. T2DM is identified as one of the major causes of premature disease, disability, and death which imposes a heavy burden on the healthcare system [2]. According to the large population studies, the effect of genetics on the pathogenesis of T2DM is estimated to be 20–25% [3–5]. Among candidate genes related to T2DM, one of the strongest genes is Transcription factor 7 like 2 (TCF7L2), which can predispose subjects to T2DM regarding the Genome-Wide Association Studies (GWAS) [4, 6]. Among different polymorphisms of the TCF7L2 gene, the T risk-allele of the rs7903146 is attributed to the strongest risk of T2DM [7]. Previous studies suggested that TCF7L2 predisposes the risk-allele carriers to T2DM through an impairment in glucagon-like peptide-1-induced insulin secretion, an impairment in β cell function, and insulin secretion, reduces insulin's ability to suppress hepatic endogenous glucose production, and the induction of insulin resistance [8–12].

To precisely examine the effect of TCF7L2, and its polymorphisms on T2DM development, understanding of modification effect of TCF7L2 on the relation between lifestyle factors and glycemic parameters is critical. Although narrative and systematic reviews have reported evidence on gene-diet interaction on T2DM [13–24], evidence for gene-diet interactions on glycemic status is scarce [25]. Some studies showed that TCF7L2 modified the relation between lifestyle factors and insulin resistance, insulin processing and secretion, insulin action, and glucose concentrations [26–29]. However, no interaction has been reported in other studies [30–34]. Therefore, we aimed to systematically review the literature that investigated the modification effect of TCF7L2 on the relation between glycemic parameters and lifestyle factors.

## Methods

The study protocol was designed as a priori and registered in the International Prospective Register of Systematic Reviews (PROSPERO) (identifier ID: CRD42020196327) and adhered to the Preferred Reporting Items for Systematic Reviews and Meta-Analyses (PRISMA) statement guidelines [35].

The Ethics Committee of the Research Institute for Endocrine Sciences, affiliated with Shahid Beheshti University of Medical Sciences (Tehran, Iran), approved the study design (IR.SBMU.ENDOCRINE.1400.104).

### Search strategy

A systematic literature search for relevant publications was performed using electronic databases, including PubMed, EMBASE, Scopus, and Web of Science, from January 1, 2000, to November 2, 2021, with no language restrictions if the abstract was published in English. Moreover, hand-searching the reference list of the eligible studies and key journals supplemented the electronic database searches. Search terms were TCF7L2, glycemic parameters, and lifestyle factors. The full details of the search strategy are shown in Table S1.

### Selection criteria

Based on the inclusion criteria, the study selection was independently done by two investigators (S.HN and S.H). Any disagreements were resolved by consultation with the third investigator (P.M). Studies were eligible to include in this systematic review if they evaluated the modification effect of TCF7L2 on the relation between glycemic parameters and lifestyle factors (diet, smoking status, and physical activity). Both observational and interventional studies were included. The exclusion criteria were as follows: 1) duplicated studies, 2) non-original papers (reviews, meta-analyses, editorials, or letters), 2) experimental studies (cell or animal studies), and 3) non-relevant articles that did not report the glycemic parameters changes by TCF7L2 genotype according to lifestyle factors. In the current study, conducting a meta-analysis was impossible because of significant heterogeneity in methodology, dietary determinants, and the study population of included studies.

### Data extraction

Two reviewers (S.HN and S.H) independently performed data extraction from the eligible studies using a standard data extraction form. Data were cross-checked, and discrepancies were handled through input from a third independent reviewer (P.M). Following items were extracted from each included study: first author’s name, year of publication, study name, country of study, study design, study population, age, gender, body mass index (BMI), the genotype of TCF7L2, number of participants, glycemic parameters, and type of intervention and duration of interventions, and outcomes. Additionally, for observational studies, follow-up duration, assessment method of lifestyle factors, and adjusted covariates were extracted.

### Quality assessment

Quality assessment of studies based on gene-lifestyle interaction on glycemic parameters was conducted based on eight items: interaction based on the primary goal, a statistical test for interaction, correction for multiple testing, correction for ethnicity, Hardy–Weinberg Equilibrium, the test of group similarity at baseline, sample size and study details [14]. The quality of randomized control trials (RCTs) was assessed using the Rob2 tool [36]. The Newcastle–Ottawa Assessment Scale (NOS) applied quality assessment for observational studies [37].

## Results

**Fig. 1:**
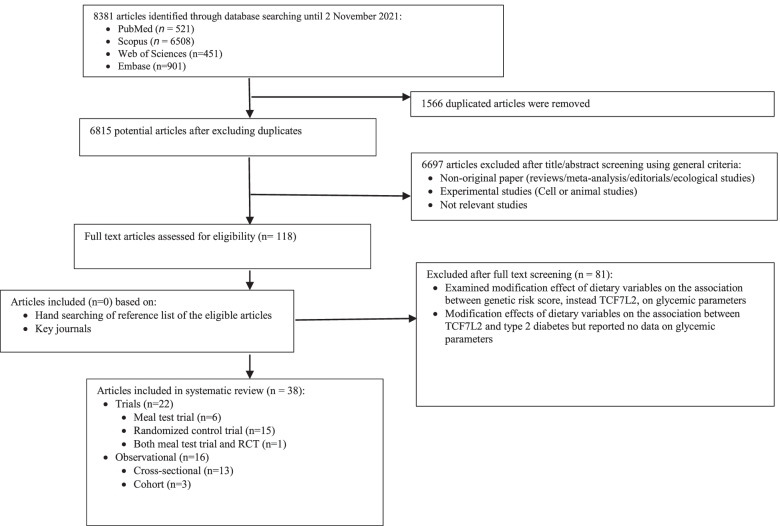
Flowchart diagram for study selection of systematic review (based on PRISMA guideline)

Figure 1 indicates the PRISMA flow diagram of the literature search and selection process. A total of 8381 articles were identified from databases (521 from PubMed, 6508 from Scopus, 901 from Embase, and 451 from Web of Sciences). All duplicated studies (1566), animal or cell studies (1097), review or editorial articles (2867), and studies not investigating the modification effect of diet on the association between TCF7L2 and glycemic parameters were excluded (2733). From the remaining 118 studies, studies that examined the modification effect of dietary variables on the association between genetic risk score, instead of TCF7L2, on glycemic parameters (*n* = 14), and studies investigated the modification effects of dietary variables on the association between TCF7L2 and T2DM but reported no data on glycemic parameters (*n* = 67) were excluded. Ultimately, 38 studies were included in the systematic review. The characteristics of the 38 studies are represented in Table 1. Out of 22 trials, six studies were meal test trials [10, 38–42], 13 studies were dietary intervention RCTs [26–28, 32–34, 43–49], two studies were physical activity RCTs [11, 50] and one study was both meal test trial and dietary intervention RCT [29]. Of 16 observational studies, 11 were nutritional cross-sectional [51–60], and prospective [61] studies and five cross-sectional and prospective studies [30, 31, 62–64] investigated the modification effect of TCF7l2 on the association between lifestyle factors (physical activity and smoking status) and glycemic parameters. The publication time ranged from 2006 to 2021.

**Table 1:** Characteristics of studies that evaluated the modification effects of TCF7L2 on the lifestyle factors and glycemic parameters

Reference	**Study type**	**Country (study name)**	**study population**	**Intervention in trial or lifestyle variables in observational studies**	**Measurement**	**Glycemic parameters**	**Mean of BMI at baseline**	**Mean of age at baseline**	**Genotype**
***Dietary variables***									
Pilgaard et al., 2009 [41]	Meal test trial	Denmark	47 young healthy men with glucose-tolerant	Standardized meals that served at 15 min (breakfast), 3 h and 15 min (lunch), 9 h (dinner), and 12 h and 30 min (sandwich), and a standardized light exercise on a bicycle was performed at 2 and 5 h	24 h profiles	glucose, insulin	23.8 in CC and 22.7 in CT/TT	18 to 23 years	rs7903146
Gjesing et al., 2011 [38]	Meal test trial	Denmark (Inter99 population-based study)	Thirty-one glucose tolerant individuals with TT genotype and 31 age- and BMI matched individual with CC genotype	A test meal consisting of 50 g white bread, 50 g black bread, 10 g butter, 40 g cheese, 20 g sugar-free jam and 200 ml milk (34% fat, 47% carbohydrate, 19% protein)	20, 10 and 0 min before and 15, 30, 45, 60, 75, 90, 105, 120, 135, 150, 180, 210 and 240 min after ingestion of the meal	Plasma glucose, serum Insulin	26.3 in CC and 25.7 in TT	53.6 in CC and 53.3 in TT	rs7903146
Perez-Martinez et al., 2012 [29]	Meal test trial	Spain	Eighty-eight healthy male with BMI < 30 kg/m2	Fatty meal (contained 65% of energy as fat, 10% of energy as protein, and 25% of energy as carbohydrates)	Before the meal and every hour until hour 6, and every 2.5 h until hour 11	HOMA-B	24.8 to 25.9 in CC, TT, CT	21.6 to 22.7 in CC, TT, CT	rs7903146
Daniele et al., 2015 [39]	Meal test trial	Italy (Genetic, physiopathology and evaluation of type 2 diabetes study)	Twenty three individuals with IFG and/or IGT and CT/TT genotype and 13 age-, gender-, and weight-matched individuals with CC genotype	A mixed meal consisting of 75 g of glucose dissolved in water spiked with 1.5 g U-13C6-glucose (CIL), 60 g of cheese, and one boiled egg	5, 15, 30, 60, 90, 120, 150, 180, and 240 min after ingestion of the meal	Glucose peak level, insulin concentration, peak insulin secretion rate, glucose sensitivity, plasma glucose levels	27.5 in CT/TT and 28.9 in CC	56.4 in CT/TT and 52.6 in CC	rs7901346
Ferreira et al., 2018 [40]	Meal test trial	Brazil	Thirty subjects with type 2 diabetes and CT/TT genotype and 26 age-, BMI, and diabetes duration matched individuals with CC genotype	500-kcal breakfast comprising 50% carbohydrates, 30% proteins, and 20% fat	at 0, 15, 30, 45, 60, 90, 120, 180, and 240 min after ingestion of meal	Plasma glucose Serum insulin and levels, HbA1c	29.8 in CT/TT 30.2 in CC	59.6 in CC 57.8 in CT/TT	rs7903146
Adamska et al., 2018 [42]	Meal test trial	polish (1000PLUS cohort)	59 men who were free from T2DM	A standardized high carbohydrate meal (450 kcal: 89% from carbohydrate, 11% from protein, 0% from fat)	30, 60, 120, 180 and 240 min after ingestion of meal	Glucose, insulin, HOMA-IR	28.3 in TT, 30.0 in CT and 28.6 in CC	39.2 in TT, 38.6 in CT and 32.0 in CC	Rs7901695, rs7903146, rs4506565
Justesen et al., 2019 [10]	Meal test trial	Denmark	40 healthy men with low birth weight and age-matched controls with normal birth weight subjects	5-day high fat overfeeding diet (50% excess energy, 60% of energy from fat) and weight maintaining 3-day control diet (35%of energy from fat)	5-day after intervention and 3-day after control diet	hepatic glucose production, peripheral insulin sensitivity, insulin stimulated glucose disposal rate, hepatic insulin resistance index, first phase insulin response	23.4 in normal birth weight and 24.8 in low birth weight (Not reported by genotype)	24 y (Not report by genotype)	rs7903146
Cauchi et al., 2008 [32]	RCT	Seven European countries: United Kingdom (England), The Netherlands, France (two centers), Spain, Czech Republic, Sweden and Denmark	Six hundred and sixty-two individuals with normal glucose tolerance and obesity	Two low calorie diets: ● Low-fat diet (20–25% of total energy from fat, 15% from protein and 60–65% from carbohydrate) ● High-fat diet (40–45% of total energy from fat, 15% from protein and 40–45% from carbohydrate)	10-week	glucose, Insulin, HOMA-IR, HOMA-β	35.7 in CC, 35.2 in CT, 35.2 in TT	20–50 year (did not report based on genotype)	rs7903146
Grau et al., 2010 [28]	RCT	Seven European countries: United Kingdom (England), The Netherlands, France (two centers), Spain, Czech Republic, Sweden and Denmark (NUGENOB study)	Six hundred and sixty-two individuals with obesity	Two low calorie diets: ● Low fat diet (20–25% of total energy from fat, 15% from protein, and 60–65% from carbohydrate) ● High fat diet (40–45% of total energy from fat, 15% from protein, and 40–45% from carbohydrate)	10 week	Fasting plasma glucose, fasting serum insulin, HOMA-IR, HOMA-B	34.4 to 36.7 in CC, CT and TT genotype in High fat and Low fat interventions groups	Not report	rs7903146
Perez-Martinez et al., 2012 [29]	RCT	Spain & Poland (LIPGENE study)	Study 1) One hundred and seventeen individuals with metabolic syndrome Study 2) Twenty elderly with non-diabetes	Study 1) Four isoenergetic diets which differed in fat quantity and quality ● A high-fat,saturated fatty acids-rich diet ● A high-fat, monounsaturated fatty acids-rich diet ● The other 2 diets were low fat, high carbohydrate diets Study 2) Three dietary intervention ● A Mediterranean diet supplemented with coenzyme Q ● Mediterranean diet not supplemented with coenzyme Q ● A Western diet rich in SFA	Study 1) 12 weeks Study 2) 4-week	HOMA-B	study 1) 34.7 in CT/TT and 34.5 in CC study 2) 30.1 in CT/TT and 33.1 CC	1) 55.6 in CT/TT and 54.2 in CC 2) 68.4 in CT/TT and 67.5 CC	rs7903146
Guevara-Cruz et al., 2012 [44]	RCT	Mexico	Thirty-two individuals with metabolic syndrome	Two low calorie diet: ● Mixture of dehydrated nopal (7 g) equivalent to 100 g of nopal, 4 g of chia seeds, 22 g of oats, 32 g of soybean protein, 0.02 g of sweetener (Splenda), and 1 g flavoring; ● 30 g of calcium caseinate, 30 g of maltodextrin, 0.02 g sweetener, and 1 g flavoring	2 month	Glucose and insulin	31.4 in intervention group and 32.6 in control group	Not reported	TCF7L2 C/T
López-Ortiz et al., 2016 [43]	RCT	Mexico	Seventy-four subjects with type 2 diabetes	Two high fiber diets: ● Nopal tortillas (equivalent to 6·2 g of fiber) ● Diet including three slices of wheat bread (equivalent to 5.5 g of fiber)	8 week	Glucose, HBA1c, insulin, HOMA-IR and HOMA-B	31 to 31.3 in both rs7903146 and rs12255372 genotype variants	51 y	rs7903146 rs12255372
Rezazadeh et al., 2018 [45]	RCT	Iran	Fifty six women with metabolic syndrome	● Four tablets of artichoke leaf extract (ALE) supplementation ● Four placebo per day	12 weeks	FBS, Insulin, HOMA_IR, Quantitative sensitivity check index (QUICKI)	among TT allele 34.7 in ALE group and 32.4 in placebo group; among C allele 36.6 in ALE group and 33.0 in placebo group	37.8 in ALE group and 39.0 in placebo group	rs7903146
Ebrahimi-Mameghani et al., 2018 [49]	RCT	Iran	Eighty women with metabolic syndrome	● 1800 mg/d of artichoke leaf extract (ALE) as four tablets ● 1800 mg/d of placebo as four tablets	12 weeks	FBS, insulin, HOMA-IR	35.3 in ALE and 33.3 in placebo groups	38.7 in ALE and 39.1 in placebo	rs7903146
Florez et al., 2006 [33]	RCT	USA (Diabetes Prevention Program)	3548 individuals with IGT and BMI ≥ 24 kg/m^2^	● Intensive lifestyle modification ● Standard care plus metformin, ● Standard care plus placebo	1 year	insulin secretion, insulin sensitivity	33.6 to 34.2 in GG, GT and TT in rs1225372 33.1 to 34.4 in CC, CT, and TT in rs7903146	50.5 to 51.3 in GG, GT and TT in rs1225372 50.6 to 51.7 in CC, CT, and TT in rs7903146	rs7903146 rs12255372
Reinehr et al., 2008 [26]	RCT	Germany (Obeldicks intervention program)	236 children with overweight	● Intervention: physical exercise, nutrition education and behavior therapy ● Controls: without any intervention	1 year	Glucose, insulin, HOMA-IR, HOMA-β, QUICKI	BMI-SDS: 2.42 in CC, 2.52 in CT, 2.55 in TT	10.8 in CC, 10.6 in CT, 10.9 in TT	rs7903146
Bo et al., 2009 [46]	RCT	Italy (Asti)	335 individuals with metabolic syndrome (139 were carrier of the CC variant and 196 were varies of the CT/TT variants)	● Intervention: A lifestyle intervention program with general recommendations carried out by trained professionals ● Control: Standard, unstructured information given by the family physician	1-y and 4-y	Glucose, Insulin, HOMA-IR and HOMA-B, IFG	Intervention group: 30.1 in CC variant and 28.8 in CT variant and 31.0 in TT variants Control group: 29.8 in CC variant, 30.1 in CT variant and 29.2 in TT variant	Intervention group: 55.5 in CC variant, 55.5 in CT variant and 56.8 in TT variants Control group: 56.3 in CC variant, 55.2 in CT variant and 55.8 in TT variant	Rs7903146
Haupt et al., 2010 [47]	RCT	Germany (Tuebingen Lifestyle Intervention Program)	309 individuals who were at risk of type 2 diabetes	● Exercise and dietary intervention The participants aimed at a weight loss of at least 5%, a reduction of caloric intake from fat of < 30% and an increase of fiber intake to at least 15 g/1000 kcal, and reduction of DFA < 10%. Individual were asked to perform at least 3 h of moderate exercise per week	9 month	Fasting glucose, glucose 120 min, insulin sensitivity,	30.3 in CC and 30.0 in CT/TT	46 in CC and 47 in Ct/TT	Rs7903146, rs12255372
McCaffery et al., 2011 [34]	RCT	USA (Diabetes prevention program)	2994 individuals that were at risk of progression to type 2 diabetes	● lifestyle intervention aiming at ≥ 7% weight loss and ≥ 150 min of physical activity per week ● Metformin 850 mg twice daily ● Placebo group	Median 2.5 year of follow-up	Insulin	34.3 in placebo, 34.0 in metformin, 34.0 in lifestyle group	50.5 y in placebo, 51.0 y in metformin, 50.7 in lifestyle group	rs7903146
Mattei et al., 2012 [27]	RCT	USA (The Preventing overweight using novel dietary strategies)	591 individuals with overweight and obese	Two low calorie diet ● Low fat (2 diets with an aim of 20% from total energy) ● High fat (2 diets with an aim of 40% from total energy)	6 month and 2 year	Glucose, insulin,	32.5 to 32.7 in rs7903146 variant and 32.1 to 32.8 in rs12255372 variant	51.6 to 52.5 in rs7903146 variant and 51.4 to 52.6 in rs12255372 variant	rs12255372 rs7903146
Walker et al., 2012 [48]	RCT	UK (RISCK study)	354 individuals who were at risk of cardiometabolic risk factors	Four isoenergetic diets: ● High monounsaturated fatty acids (MUFA)/high glycemic index (GI) ● High MUFA/low GI ● Low fat /high GI ● LF/low GI	24 weeks	AIRg (actue insulin secretion), deposition index, insulin sensitivity	28.7 for total population	53.5 for total population	rs7901695
Ruchat et al., 2009 [51]	Cross-sectional	Canada (Quebec Family Study)	669 adults with non-diabetes	Dietary fatty acid (3-day (2 week days, 1 weekend day))	––-	Fasting glucose, HOMA-IR, HOMA-B, insulin secretion, Two hour glucose, The Cederholm index (adjusted for age and sex)	27.7 in total population	40.5 in total population	rs12573128 rs10128255 rs7903146 rs17685538 rs11196205 rs11196203 rs4918789 rs3750804 rs3750805 rs176632 rs11594610 rs1885510 rs7901695
Nettleton et al., 2010 [52]	cross-sectional	Europe (14 cohort study)	48,000 participants with non-diabetes	Whole grain (FFQ (11 cohorts) a lifestyle questionnaire (1 cohort) multiple 24-h recalls (1 cohort) 7-day dietary diaries (1 cohort))	–-	Fasting glucose and fasting insulin (adjusted for Age, gender, energy intake and center)	From 20.0 to 29.7 in different cohort studies	From 11.2 to 76.4 in different cohort studies	Rs4506565
Delgado-Lista et al., 2011 [53]	cross-sectional	Ireland, UK, Norway, France, The Netherlands, Spain, Poland and Sweden (LIPGENE dietary intervention study)	450 participants with non-diabetes	Plasma saturated fatty acids concentration	–-	insulin, glucose, HOMA-IR, HOMA-β, acute insulin response to glucose (AIRg)	32.6 in CC, 32.3 in CT, 32.5 in TT	53.7 in CC, 54.9 in CT, 55.3 in TT	rs12255372 rs4506565 rs7901695 rs7903146 rs17685538 rs290481 rs11196224 rs3814573 rs6585196 rs1885510
Phillips et al., 2012 [61]	Prospective case control study with 7.5 year follow-up	France (LIPGENE)	964 participants (participants with Metabolic syndrome who were matched participants with non-metabolic syndrome)	Dietary fatty acid (food frequency questionnaire)	–-	Fasting glucose, insulin, HOMA-IR, QUICKI (adjusted for Age, gender, BMI, smoking status, energy intake, physical activity and medication use)	25.0 to 26.2 in CC, CT and TT	57.9 to 58.3 in CC, CT and TT	rs7903146
Hindy et al., 2012 [54]	Cross sectional	Sweden (The Malmö Diet and Cancer Study ( MDCS))	5216 participants with non-diabetes	Dietary fiber (a 7-day menu book where lunch, dinner meals and cold beverages, including alcohol, were recorded; and a dietary 168-item questionnaire)	–-	HBA1c, fasting glucose	25.5 to 25.7 in CC, CT and TT (Age, gender, BMI, total energy intake, season and method)	58.0 to 58.1 in CC, CT and TT	Rs7903146
Corella et al., 2013 [55]	cross-sectional	Spain (the PREvención con DIetaMEDiterr_anea (PREDIMED))	7018 patients with type 2 diabetes or participants at high risk of cardiovascular risk factors	Mediterranean dietary pattern (food frequency questionnaire)	–-	Fasting glucose concentrations (adjusted for age, sex, BMI, type 2 diabetes, total energy intake, alcohol consumption, smoking, physical activity, medication)	30.0 in total population	67.0 in total population	rs7903146
Ouhaibi-Djellouli et al., 2014 [56]	Cross sectional	Algeria (Insulino-résistance à Oran (ISOR))	720 participants (both diabetic and non-diabetes participants)	Milk and dessert (food frequency questionnaire)	–-	Glucose, insulin, HOMA-IR, HOMA-B (adjusted for age, gender, smoking status, physical activity, BMI)	26.4 in CC, 25.7 in CT, 24.9 in TT	42.8 in non-T2D and 52.0 in T2D subjects	rs7903146
Lu et al., 2017 [57]	cross sectional	USA	120 patients with non-diabetes	Free fatty acid concentration	––	HOMA-IR (adjusted for Age, gender, BMI)	27.4 in CC and 27.3 in TT	41 in CC and 42 in TT	rs7903146
Bodhini et al., 2017 [58]	Cross sectional	India (Chennai Urban Rural Epidemiology Study (CURES))	1681 participants (821 normal glucose tolerance and 861 participants with diabetes)	Macronutrient and dietary fiber (food frequency questionnaire)	–-	Fasting plasma glucose (adjusted for age, gender, BMI, energy intake)	23.6 in normal glucose tolerant and 25.3 in type 2 diabetes participants	41.3 in normal glucose tolerant and 50.5 in type 2 diabetes participants	rs12255372 rs7903146
Barabash et al., 2020 [59]	Cross sectional	Spain (St Carlos GDM prevention study)	874 pregnant women	Mediterranean dietary pattern (food frequency questionnaire)	––	Fasting blood glucose (Ethnicity, age, parity, family history of diabetes and BMI)	23.3 to 24.3 based on adherence to Mediterranean diet	31.2 to 33.9 based on adherence to Mediterranean diet	rs7903146
Bauer et al., 2021 [60]	Cross sectional	Poland	810 subjects with non-diabetes	Macronutrient intake (3-day food diaries)	––-	Fasting plasma glucose, Insulin concentrations, HbA1c, HOMA-IR, HOMA-β (adjusted for Age, gender, BMI, energy intake, physical activity levels)	28.7 in TT, 28.2 in CT and 28.3 in CC	40.9 in TT, 40.9 in CT, and 40.8 in CC	rs7901695
***Physical activity***									
Ruchat et al., 2010 [50]	Trial	United States and Canada ( HEealth, Risk factors, exercise Training, AND Genetics (HERITAGE))	481 participants without of chronic diseases	Exercise Program (three times per week)	20-week	Fasting glucose, Fasting insulin insulin sensitivity index, acute insulin response to glucose, disposition index, glucose effectiveness	25.8 kg/m^2^	35.9 y	rs4903146
Alibegovic et al., 2010 [11]	Trial	Denmark	38 healthy young Caucasian men	Bed rest	9 days	Insulin, glucose, Intravenous glucose tolerance test (β-cell test) first-phase insulin response,, second-phase insulin secretion	23.0 in TT/CT and 24.4 in CC	25.6 in TT/CT and 25.2 in CC	rs7903146
Brito et al., 2009 [62]	Cohort	Sweden (Malmo¨ Preventive Project)	16,003 individuals at high risk of developing chronic disease	Physical activity that assessed using computer-based questionnaire	16 years’ mean follow-up time	Impaired glucose regulation, 2-h plasma glucose	24.6 in physical inactive and 24.2 in physical active participants	44.7 in physical inactive and 45.7 in physical active participants	rs7903146
Scott et al., 2012 [30]	All data were cross-sectional except for atherosclerosis Risk in Communities Study (ARIC) where PA data were available at the visit 3 years before 2-h glucose measurement	USA, Finland, Switzerland, UK, Sweden, Denmark, Europe, German, British (Meta-analyses of glucose and insulin related traits consortium (MAGIC))	48,362 individuals with Non-diabetes and BMI ≥ 18.5 kg/m^2^	Physical activity that assessed using Different questionnaire	––-	2-h glucose	Not reported	Not reported	Rs12243326
Jung et al., 2016 [64]	Cross sectional	Korean (Genomics and Randomized Trials Network (GARNET))	1027 postmenopausal women	Physical activity that assessed using questionnaire, dietary intake by FFQ	–-	Insulin, fasting glucose, HOMA-IR	Not reported	63 to 65 in obese and non-obese women, respectively	Rs4506565
***Smoking status***									
Wu et al., 2020 [31]	Cohort	Europe and Africa (The Atherosclerosis Risk in Communities Study (ARIC), the Coronary Artery Risk Development in Young Adults Study (CARDIA), the Cardiovascular Health Study (CHS), the Framingham Heart Study (FHS), and the Multi-Ethnic Study of Atherosclerosis (MESA))	97,773 participants with non-diabetes	Smoking status: ● Current or former smokers at baseline (ever smokers) ● No current or past smoking history (never smokers)	Not reported	Fasting glucose	23.9 to 40.8 in different cohorts	25.6 to 76.9 in different cohorts	rs4132670 rs12243326
Lin, 2020 [63]	Cross sectional	Taiwan	25,460 participants aged 30–70 year	Smoking status: ● Smokers Nonsmokers	––	Fasting glucose HbA1c	25.4 in smokers and 24.2 in nonsmokers	46.4 in smokers and 49.2 in nonsmokers	rs4132670 rs12243326

### Characteristics of studies

#### Meal test trials

Of the seven studies included, six studies were done in Europe [10, 29, 38, 39, 41, 42] and one study in Brazil [40]. The most frequently studied variant was rs7903146 [10, 29, 38–42]. Subjects were healthy males [10, 29, 38, 41], males with non-diabetes [42], participants with impaired fasting glucose (IFG) or impaired glucose tolerance (IGT) who were at risk of developing T2DM [39], and subjects with T2DM with disease duration < 10 years [40]. Four studies were conducted on subjects with BMI ≥ 25 kg/m^2^ [38–40, 42] and others on subjects with BMI < 25 [10, 41]. The dietary interventions included a standardized high carbohydrate meal (89% carbohydrate, 11% protein, and 0% fat) [42], 500 cal breakfast (50% carbohydrate, 30% protein, and 20% fat) [40], a test meal consisting of 50 g white bread, 50 g black bread, 10 g butter, 40 g cheese, 20 g sugar-free jam and 200 ml milk (47% carbohydrate, 19% protein, and 34% fat) [38], a standard meal test (25% carbohydrate, 10% protein, and 65% fat) [29], high fat overfeeding diet (50% excess energy, 60% fat) [10], and standard mixed meal consisting 75 g of glucose, 60 g of cheese and one boiled egg [39]. Only one study investigated the effects of standard meals and physical activity, including light bicycle exercise [41].

#### Dietary intervention RCTs

Of 14 studies, seven were conducted in Europe [26, 28, 29, 32, 46–48], three in the USA [27, 33, 34], two in Mexico [43, 44], and two in Iran [45, 49]. The most frequently studied variant was rs7903146 [26–28, 32–34, 43–47]. Trials were conducted on participants with impaired glucose tolerance (IGT) (FPG of < 125 mg/dL and a 2-h post-load plasma glucose 140 to 199 mg/dL, which is measured during a 75-g oral glucose load) [33], adult participants with obesity [27, 28, 32], children with overweight [26], participants with T2DM [43], and metabolic syndrome [29, 44–46, 49], elderly participants aged over 65 years [29], and participants who were at risk of developing T2DM (based on impaired glucose tolerance, diagnosis of gestational diabetes, diagnosis of the polycystic ovarian syndrome, atherogenic lipoprotein phenotype, BMI ≥ 24, had a family history of diabetes) [34, 47, 48]. Studies in Europe used low fat (20–25%) and high fat (40–45%) hypo-energetic diet (-600 kcal/day) [32], high saturated fatty acids (SFA) and high glycemic index (SFA, 18% energy; monounsaturated fatty acids (MUFA), 12% energy), high mono-unsaturated fatty acids (MUFA)/high glycemic index (MUFA, 20% energy; SFA, 10% energy), high MUFA/low glucemic ndex (SFA, 10% energy; MUFA, 11% energy), low fat/high glycemic index (SFA, 10% energy; MUFA, 11% energy), and low fat/low glycemic index (SFA, 10% energy; MUFA, 11% energy) [48], low fat (20–25% of energy from fat, 15% from protein, and 60–65% from carbohydrate) and high fat diet ((40–45% of energy from fat, 15% from protein, and 40–45% from carbohydrate) hypocaloric diet (-600 kcal/day) [28], and high fat/high SFA (16% from SFA), high fat/high MUFA (20% from MUFA), low fat/high carbohydrate (28% from fat and included a 1.24 g/d supplement from PUFA capsules), low fat/high carbohydrate (28% from fat and included a 1.24 g/d supplement from sunflower seed oil capsules) [29].

Studies in Mexico used a dietary pattern that included nopal, chia seeds, oats, and soybean protein as a food rich in fiber [44], and dietary interventions in which intake of fiber was from nopal tortillas or wheat bread [43], and Iranian studies used artichoke leaf extract supplementation [45, 49]. Others investigated the intensive lifestyle modifications [26, 27, 33, 34, 46, 47]. Intensive lifestyle intervention included the lifestyle intervention aiming for ≥ 7% weight loss and ≥ 150 min of physical activity per week during 2.5 years of follow-up [33, 34], ≥ 5% weight loss, reduction of caloric intake from fat to < 30% and an increase of fiber intake to at least 15 g/1000 kcal and ≥ 3 h of moderate physical activity per week during the 2-year intervention [47], the general recommendation-based program of lifestyle intervention carried out by trained professionals versus standard unstructured information given by family physicians during the 1-y intervention [46], physical exercise, nutrition education, and behavioral therapy, including the individual psychological care during the 1-year intervention [26], low-fat diet (20% from total energy) and high-fat diet (40% from total energy) hypocaloric diet (-750 kcal) during two years [27]. The trials’ sample size ranged from 20 [29] to 3548 [33] subjects, with a mean age of 20 to 67 years.

#### Nutritional observational studies

The characteristics of the 11 included observational studies are shown in Table 1. All studies were cross-sectional except one that was cohort with a 7.5-year follow-up [61]. Seven studies were carried out in Europe [52–55, 59–61], and others were in Canada [51], Algeria [56], the USA [57], and India [58]. Rs7903146 was the most important studied variant [51, 53–59, 61]. Ten studies included both biological sex, and only one study was done on women with gestational diabetes mellitus [59]. The number of participants ranged from 120 [57] to 48,000 [52]. Different methods were used to assess the dietary intake, including self-reported measurements (food frequency questionnaire [52, 55, 56, 58, 59, 61], 3-day food diaries [51, 60], and 7-day dietary recall [54] and biomarkers (plasma fatty acids) [53, 57].

#### Other lifestyle (smoking and physical activity) observational and clinical trials studies

The study characteristics are shown in Table 1. Five studies were observational [30, 31, 62–64], and two were clinical trials [11, 50]. Most studies were carried out in Europe [11, 30, 62], and others were conducted in the USA and Canada [50], Korea [64], Europe and Africa [31], and Taiwan [63]. Five studies investigated whether different variants of the TCF7L2 gene modify the association between physical activity and glycemic homeostasis [11, 30, 50, 62, 64]. Only two studies investigated the modulation effects of rs4132670 and rs12243326 on the association between smoking status and glycemic parameters [31, 63]. Most studies included both biological sex. The average BMI and age range were 23.0 to 40.8 kg/m^2^ and 25.6 to 76.9 years, respectively.

#### Methodological quality assessment

Among 38 studies assessed for their methodological quality in gen-lifestyle interaction effects on glycemic parameters, 11 studies had high, 25 had intermediate, and two had poor quality. Small sample size, missing information for the similarity between participants with risk and non-risk allele at baseline, and no correction for multiple testing often reduced methodological quality (Table S2).

Among 14 RCTs, ten studies met all the criteria for methodological quality assessment according to the Rob2 tool. Three studies were considered of some concern, and one study was considered high-risk (Table S3). According to NOS, the observational studies were considered good and very good (Tables S4, and S5).

### Main finding

#### Meal test trials

The effect of TCF7L2 rs7903146 on glycemic parameters following a standardized test meal is contradictory. Among healthy glucose tolerant individuals, a standard test meal includes 50 g white bread, 50 g black bread, 10 g butter, 40 g cheese, 20 g sugar-free jam, and 200 ml milk (47% carbohydrate, 19% protein, and 34% fat) [38], high-fat diet including 65% fat, 10% protein and 25% carbohydrate [29], high fat overfeeding diet (50% excess energy, 60% fat) [10], and high carbohydrate test meal (89% carbohydrate, 11% protein and 0% fat) [42] increased glucose concentration among TT allele carriers. Only one study reported no effect of rs7903146 on plasma glucose after standard meal ingestion, which included standard breakfast, lunch, dinner, and a standardized light exercise [41]. Among IFG participants, consumption of standard meals (consisting of 75 g glucose, 60 g cheese, and one boiled egg) reduced plasma glucose peak levels in T-carriers [39], but in patients with T2DM, no differences in fasting plasma glucose were observed in both the CC and CT/TT groups, after meal test which consisted of 50% carbohydrate, 30% protein and 20% fat [40].

No difference in insulin concentration was shown among healthy glucose tolerant individuals with risk and non-risk alleles in response to the ingestion of standard meals with different contents of macronutrients (47–89% carbohydrate, 11–19% protein, 0–34% fat) [38, 41, 42]. In contrast, plasma insulin concentrations were significantly higher in the T-carrying group after the ingestion of standard meals in impaired fasting glucose (IFG), impaired glucose tolerance (IGT), and type 2 diabetes participants [39, 40].

In glucose tolerance individuals, insulin resistance decreased more among C than T allele carriers [10, 42], but in IFG and/or IGT participants, no difference in glucose sensitivity was observed between the risk and non-risk alleles [39]. Among healthy glucose tolerant males, β-cell dysfunction was reduced among T allele carriers after ingestion of a standard meal which included standard breakfast, lunch, dinner, and a standardized light exercise [41], but beta-cell dysfunction did not differ between risk and non-risk allele carriers after ingestion of high-fat diet with 60–65% fat [10, 29].

#### Dietary intervention RCTs

Findings from studies on participants at risk of T2DM and participants with metabolic syndrome and T2DM reported that TCF7L2 variants did not modulate the effect of dietary interventions on glycemic parameters [29, 33, 34, 43, 46–48]. In Diabetes Prevention Program (DDP) and Diabetes Prevention Program Outcomes Study (DPPOS), in response to lifestyle modification, no difference in insulin concentration, insulin-secretion, or insulin-sensitivity indices was observed by rs7903146 and rs12255372 over a one-year follow-up among participants who were at risk of progression to T2DM [33, 34]. In the RISK study, TCF7L2 SNP rs7901695 did not modulate the effect of dietary interventions on the change of acute insulin secretion, insulin sensitivity, and deposition index (a measure of the beta cell's ability to compensate for changes in insulin resistance) during 24 weeks of intervention in participants who were at risk of T2DM [48]. In Tuebingen Lifestyle Intervention Program (TULIP), during a 9-month exercise and dietary intervention, no significant effects of rs11196205 and rs7895340 on glucose changes, 2-h glucose, insulin sensitivity, and insulin secretion were observed among participants who were at risk of type 2 diabetes [47]. Among subject with risk or non-risk allele of rs7903146 with metabolic syndrome, there was no differences in insulin, homeostatic model assessment of β-cell function (HOMA-β), and the homeostasis model assessment-estimated insulin resistance (HOMA-IR) after general recommendations regarding healthy diet, physical activity, and behavior modifications that given by trained professionals [46].

#### Homeostatic model assessment

Regarding the weight loss dietary interventions with calorie restriction to 500- 600 kcal/day, three studies using hypocaloric diets for 8 to 10 weeks reported no significant effect of TCF7L2 rs7903146 on fasting glucose, insulin concentrations, insulin secretion, insulin resistance, and HOMA-β in overweight, obese, and metabolic syndrome subjects [28, 32, 44]. However, in long-term weight loss dietary interventions, individuals with non-diabetes, overweight and obese, and rs12255372 risk genotype had greater decreases in glucose and insulin concentrations per unit reduction in BMI compared to the non-risk allele [27]. In addition, lifestyle interventions among overweight children showed that improvement in insulin resistance was lower among T allele carriers [26].

Regarding the artichoke extract supplementation (ALE), two studies observed that ALE supplementation significantly decreased the insulin concentration and HOMA-IR in the TT genotype of the rs7903146 variant of TCF7L2 [45, 49]. There were no significant differences between the groups in TCF7L2 rs790316 variants in response to ALE supplementation [45].

#### Nutritional observational studies

The most commonly investigated dietary exposure was dietary fat intake (total dietary fat and SFA) and plasma fatty acids concentrations [51, 53, 57, 58, 61], followed by protein, carbohydrate, dietary fiber, whole grains, milk, desserts [52, 54, 56, 60] and Mediterranean dietary pattern [55, 59]. In a Quebec family study, among different variants of the TCF7L2 gene, the rs12573128 genotype modified the association between total dietary fat intake and glycemic parameters; values of insulin sensitivity and glucose tolerance were higher among carriers of the rs12573128 A/A genotype with lower, but not higher, total dietary fat intake [51]. In the LIPGENE study, during a 7.5-year follow-up, high intake of SFA was associated with impairment of insulin sensitivity and higher insulin concentrations in the T-risk allele of rs7903146, compared to the non-risk allele [61]. Among subjects with high concentrations of SFA and free fatty acid (FFA), insulin concentration and HOMA-IR were higher in the TT rs11196224, GA/AA rs290481, and TT rs7903146 compared to the wild-type allele [53, 57]. However, in Chennai Urban Rural Epidemiology Study (CURES), no interaction was found between rs12255372 and rs7901695 and total dietary fat intake on fasting blood glucose [58, 60], hemoglobin A1c (HbA1c), HOMA-IR, and HOMA-β [60]. Furthermore, the TCF7L2 rs7903146 variant modified the association between consumption of dietary fiber; and dessert, but not milk, and glycemic parameters [54, 56]. The CC genotype carriers, but not the TT genotype, had lower HbA1c levels with higher fiber intake [54], and consumption of one dessert/day was associated with higher fasting plasma glucose concentrations in rs7903146 T allele carriers [56]. However, in a meta-analysis of 14 cohorts, no interaction was observed between glucose and insulin concentrations, rs4506565, and whole grains [52]. In addition, adherence to the Mediterranean dietary pattern modified the effect of rs7903146 polymorphism on glucose concentration. In low adherence levels, glucose concentration was higher in TT individuals; compared to CT/CC. However, in high adherence, no difference in glucose concentration was found between individuals with risk and non-risk alleles [55, 59].

#### Other lifestyle (smoking and physical activity) observational studies

The modification effect of TCF7L2 variants on the effect of physical activity levels on glycemic parameters was contradictory. The TCF7L2 rs4506565 T-allele tends to positively associate with glucose levels, insulin concentrations, and HOMA-IR in participants with low, but not high, physical activity levels [64]. In contrast, the rs7903146 T allele was associated with impaired glucose regulation and 2-h glucose in the active participants [62]. In addition, in response to bed-rest, insulin concentrations and insulin secretion were significantly lower in rs7903146 TT/CT genotype compared to the CC genotype [11]. Furthermore, an interaction was seen between two single nucleotide polymorphisms (SNPs) (rs4132670 and rs12243326) and smoking on HbA1c and fasting blood glucose in the active smoking participants [63]. However, three studies observed no effects of rs12243326 and rs7903146 on glycemic parameters in participants with different levels of physical activity [30, 50] and smoking status [31].

## Discussion

This study systematically reviewed 38 articles on the modification effect of TCF7L2 on the relation between lifestyle and glycemic parameters. In the current systematic review, observational studies showed that TCF7L2 modified the association between the diet (including dietary and serum fatty acids and fiber) and insulin resistance. In contrast, the effect of this gene on other glycemic parameters, including glucose and insulin concentrations, was inconsistent. Most observational studies had been conducted on participants with non-diabetes showing that TCF7L2 modified the association between diet (fatty acids and fiber) and insulin resistance. In addition, findings from meal test trials showed that among healthy risk allele carriers, consumption of meals with different percentages of total dietary fat, increased glucose concentrations, and impaired insulin resistance compared to non-risk allele carriers. However, ten randomized controlled trials with an intervention period of fewer than ten weeks and more than one year showed that TCF7L2 did not modify glycemic parameters in response to a dietary intervention involving different macronutrients. However, two weight loss dietary interventions with a duration > one year showed an improvement in insulin resistance and a decreases in glucose and insulin concentrations in non-risk allele subjects with overweight/obesity. Two RCTs observed that ALE supplementation significantly decreased insulin concentration and HOMA-IR in the TT genotype of the rs7903146 variant of TCF7L2. Four studies suggest that physical activity levels and smoking status modified the association between TCF7L2 and glycemic parameters. However, three studies observed no effect of rs12243326 and rs7903146 on glycemic parameters in participants with different levels of physical activity and smoking status.

The discrepancy between the findings of observational studies and trials may be due to differences in the study population, dietary determinants, and weight change. Most included observational studies had been conducted among subjects with non-diabetes, and in most of them, insulin resistance was further impaired with high consumption of fatty acids or high concentration of plasma fatty acids in risk allele carriers of TCF7L2 [51, 53, 57, 61]. Although previous studies have suggested that impairment in β-cell function predisposes the risk-allele carriers of the TCF7L2 variants to the progression of T2DM [8, 9], the dysfunction in β-cell may be due to the insulin resistance that is more pronounced in healthy T-allele risk carriers [10, 11]. There is evidence that participants with a family history of diabetes and genetic background of T2DM responded differentially to dietary and pharmacological treatment [65, 66]. Regarding the metformin treatment, in participants with a new diagnosis of T2DM, insulin resistance decreased more among T-allele carriers. However, this response became less efficacious among participants with the progression of the disease [65]. In addition, the dietary intervention had little effect on the prevention and delay in initiating glucose-lowering treatment in subjects with a family history of T2DM and those with high hepatic insulin resistance and β-cell dysfunction [66]. Moreover, in observational studies, the effect of fatty acids (both diet and plasma) on the relationship between TCF7L2 and glycemic parameters has been more studied [51, 53, 57, 61]. Fatty acids induce insulin resistance [67, 68], and this effect was more pronounced in TCF7L2 risk-allele carriers [51, 53, 57, 61].

In line with the observational studies, findings from meal test trials showed that among healthy risk allele carriers, consumption of high-fat meals, increased hepatic production of glucose, serum glucose concentrations, and impaired insulin resistance, compared to non-risk allele carriers [10, 38, 40, 42]. However, most RCTs showed that TCF7L2 did not modify glycemic parameters in response to dietary interventions [28, 29, 34, 47, 50]. This discrepancy can be due to several reasons. First, the target population in randomized trials were overweight and obese subjects who were predisposed to insulin resistance and T2DM. As mentioned above, the difference in response to treatment was reported between the TCF7L2 risk allele and the non-risk allele in the early but not in the late stage of diabetes [65, 66]. Second, the influence of TCF7L2 on glycemic parameters can be modified by weight loss. In two trials, weight loss led to better glycemic control in the TCF7L2 risk genotype compared to the non-risk genotype [26, 27]. However, in other trials, no influence of this gene on glycemic parameters happened, along with any change in weight during dietary interventions [28, 29, 34, 47, 50]. This may be due to the that TCF7L2 also regulates adipose tissue via the Wnt pathway, and a potential association has been suggested between TCF7L2 and obesity development [67]. Third, macronutrient distribution, depending on TCF7l2 genotype, may also influence improvement in cardiometabolic risk factors [69]. In the POUNDS LOST and NUGENOB studies, a more significant reduction in weight, waist circumference, and insulin resistance was documented in response to a low-fat diet, but not a high-fat diet, in individuals with risk alleles of rs12255372 and rs7903146 genotypes [27, 28]. This finding aligns with observational studies, which showed that the TCF7L2 might interact with fatty acids on insulin resistance status [51, 53, 57, 61]. Future observational cohort research and randomized controlled trials on TCF7L2-diet interaction on glycemic parameters can provide opportunities to understand the exact mechanism of this gene and whether this information leads to determining effective strategies for the prevention and management of T2DM.

In our systematic review, the methodological quality of included observational studies was intermediate and high. In most of these studies, the modification effect of TCF7L2 on diet and glycemic parameters had been assessed as the primary outcome, and multiple testing has been controlled, Hardy Weinberg reported, finding adjusted for BMI, and dietary variables had been assessed using valid and reliable FFQs. However, most of these observational studies were cross-sectional, which cannot prove causality, and had been conducted in Europe, which limits generalizability to other countries. Also, these studies included subjects with non-diabetes that cannot be extrapolated to other subjects, such as T2DM. In addition, despite the high quality of methodology in trials, interpretation of findings should be made with caution because most RCTs were not primarily designed for this purpose; therefore, their findings were reported based on post-hoc analysis, and subjects did not stratify based on TCF7L2 genotypes, the accuracy of diet assessment in the evaluation of adherence to interventions was limited, and the sample size was small. Moreover, regarding the great heterogeneity in methodology, dietary determinants, and study population conducting a meta-analysis was impossible.

## Conclusion

To date, limited studies have been conducted on the modification effect of TCF7L2 on lifestyle factors to improve glycemic parameters. In the current study, the modification effects of TCF7L2 on the relation between the dietary intervention and glycemic parameters were observed in observational studies and weight loss RCTs. Weight can play an important role in the modification effect of this gene on the relationship between dietary factors and glycemic parameters. In addition, the modification effects of TCF7L2 on the relation between the lifestyle factors (physical activity and smoking status) and glycemic parameters were contradictory.

## Supplementary Information

**Additional file 1: Table S1. **Details of the search strategy in electronic databases. **Table S2.** Quality assessment of studies based on gene-lifestyle interaction on glycaemic parameters. **Table S3.** Quality assessment of cohort studies by using the Rob2 tool. **Table S4.** Quality assessment of cross-sectional studies by using the Newcastle Ottawa Scale. **Table S5.** Quality assessment of cohort studies by using the Newcastle Ottawa Scale

**Additional file 2.** 

**Additional file 3.** 

**Additional file 4.** 

## Data Availability

Not applicable.

## Abbreviations

GWAS: Genome-Wide Association Studies
TCF7L2: Transcription factor 7 like 2
PROSPERO: The International Prospective Register of Systematic Reviews
PRISMA: Preferred Reporting Items for Systematic Reviews and Meta-Analyses
RCTs: Randomized control trials
NOS: The Newcastle–Ottawa Assessment Scale
IFG: Impaired fasting glucose
IGT: Impaired glucose tolerance
DPPOS: Diabetes Prevention Program Outcomes Study
DDP: Diabetes Prevention Program
TULIP: Tuebingen Lifestyle Intervention Program
HOMA-β: Homeostatic model assessment of β-cell function
HOMA-IR: The homeostasis model assessment-estimated insulin resistance
ALE: Artichoke extract supplementation
SFA: Saturated fatty acid
FFA: Free fatty acid
CURES: Chennai Urban Rural Epidemiology Study
HbA1c: Hemoglobin A1c
SNPs: Single nucleotide polymorphisms

## References

[1] Saeedi P, Petersohn I, Salpea P, Malanda B, Karuranga S, Unwin N (2019). Global and regional diabetes prevalence estimates for 2019 and projections for 2030 and 2045: Results from the International Diabetes Federation Diabetes Atlas, 9(th) edition. Diabetes Res Clin Prac.

[2] Dal Canto E, Ceriello A, Rydén L, Ferrini M, Hansen TB, Schnell O (2019). Diabetes as a cardiovascular risk factor: An overview of global trends of macro and micro vascular complications. Eur J Prev Cardiol.

[3] Doria A, Patti ME, Kahn CR (2008). The emerging genetic architecture of type 2 diabetes. Cell Metab.

[4] Grant SF, Thorleifsson G, Reynisdottir I, Benediktsson R, Manolescu A, Sainz J (2006). Variant of transcription factor 7-like 2 (TCF7L2) gene confers risk of type 2 diabetes. Nat genet.

[5] Altshuler D, Hirschhorn JN, Klannemark M, Lindgren CM, Vohl MC, Nemesh J (2000). The common PPARgamma Pro12Ala polymorphism is associated with decreased risk of type 2 diabetes. Nat genet.

[6] Sladek R, Rocheleau G, Rung J, Dina C, Shen L, Serre D (2007). A genome-wide association study identifies novel risk loci for type 2 diabetes. Nature.

[7] Zeggini E, Scott LJ, Saxena R, Voight BF, Marchini JL, Hu T (2008). Meta-analysis of genome-wide association data and large-scale replication identifies additional susceptibility loci for type 2 diabetes. Nat Genet.

[8] Cropano C, Santoro N (2017). The rs7903146 Variant in the TCF7L2 Gene Increases the Risk of Prediabetes/Type 2 Diabetes in Obese Adolescents by Impairing β-Cell Function and Hepatic Insulin Sensitivity. Diabetes Care.

[9] Chen J, Ning C, Mu J, Li D, Ma Y, Meng X (2021). Role of Wnt signaling pathways in type 2 diabetes mellitus. Mol Cell Biochem.

[10] Justesen L, Ribel-Madsen R, Gillberg L, Hansen NS, Wulff AL, Grunnet LG (2019). TCF7L2 Expression Is Regulated by Cell Differentiation and Overfeeding in Human Adipose Tissue. Endocr Res.

[11] Alibegovic AC, Sonne MP, Højbjerre L, Hansen T, Pedersen O, van Hall G (2010). The T-allele of TCF7L2 rs7903146 associates with a reduced compensation of insulin secretion for insulin resistance induced by 9 days of bed rest. Diabetes.

[12] Villareal DT, Robertson H, Bell GI, Patterson BW, Tran H, Wice B (2010). TCF7L2 variant rs7903146 affects the risk of type 2 diabetes by modulating incretin action. Diabetes.

[13] Franks PW, Mesa JL, Harding AH, Wareham NJ (2007). Gene-lifestyle interaction on risk of type 2 diabetes. Nutr Metab Cardiovasc Dis.

[14] Dietrich S, Jacobs S, Zheng JS, Meidtner K, Schwingshackl L (2019). Gene-lifestyle interaction on risk of type 2 diabetes: A systematic review. Obes Rev.

[15] Franks PW, Merino J (2018). Gene-lifestyle interplay in type 2 diabetes. Curr Opin Genet Dev.

[16] Franks PW, Paré G (2016). Putting the Genome in Context: Gene-Environment Interactions in Type 2 Diabetes. Curr Diab Rep.

[17] Franks PW (2012). The complex interplay of genetic and lifestyle risk factors in type 2 diabetes: an overview. Scientifica (Cairo).

[18] Temelkova-Kurktschiev T, Stefanov T (2012). Lifestyle and genetics in obesity and type 2 diabetes. Exp Clin Endocrinol Diabetes.

[19] Weyrich P, Stefan N, Häring HU, Laakso M, Fritsche A (2007). Effect of genotype on success of lifestyle intervention in subjects at risk for type 2 diabetes. J Mol Med (Ber).

[20] Cornelis MC, Hu FB (2012). Gene-environment interactions in the development of type 2 diabetes: recent progress and continuing challenges. Annu Rev Nutr.

[21] Franks PW, Pearson E, Florez JC (2013). Gene-environment and gene-treatment interactions in type 2 diabetes: Progress, pitfalls, and prospects. Diabetes Care.

[22] Ortega Á, Berná G, Rojas A, Martín F, Soria B (2017). Gene-Diet Interactions in Type 2 Diabetes: The Chicken and Egg Debate. Int J Mol Sci.

[23] Li SX, Imamura F, Ye Z, Schulze MB, Zheng J, Ardanaz E (2017). Interaction between genes and macronutrient intake on the risk of developing type 2 diabetes: systematic review and findings from European Prospective Investigation into Cancer (EPIC)-InterAct. Am J Clin Nutr.

[24] Asif S, Morrow NM, Mulvihill EE, Kim KH (2020). Understanding Dietary Intervention-Mediated Epigenetic Modifications in Metabolic Diseases. Front Genet.

[25] Parastouei K, Rostami H, Ramezani AA, Tavakoli H, Alipour M (2020). Gene-diet interaction of FTO-rs9939609 gene variant and hypocaloric diet on glycemic control in overweight and obese adults: a systematic review and meta-analysis of clinical trials. Chin Med J (Engl).

[26] Reinehr T, Friedel S, Mueller TD, Toschke AM, Hebebrand J, Hinney A (2008). Evidence for an influence of TCF7L2 polymorphism rs7903146 on insulin resistance and sensitivity indices in overweight children and adolescents during a lifestyle intervention. Int J Obes (Lond).

[27] Mattei J, Qi Q, Hu FB, Sacks FM, Qi L (2012). TCF7L2 genetic variants modulate the effect of dietary fat intake on changes in body composition during a weight-loss intervention. Am J Clin Nutr.

[28] Grau K, Cauchi S, Holst C, Astrup A, Martinez JA, Saris WH (2010). TCF7L2 rs7903146-macronutrient interaction in obese individuals' responses to a 10-wk randomized hypoenergetic diet. Am J Clin Nutr.

[29] Perez-Martinez P, Perez-Caballero AI, Garcia-Rios A, Yubero-Serrano EM, Camargo A, Gomez-Luna MJ (2012). Effects of rs7903146 variation in the Tcf7l2 gene in the lipid metabolism of three different populations. PLoS ONE.

[30] Scott RA, Chu AY, Grarup N, Manning AK, Hivert MF, Shungin D (2012). No interactions between previously associated 2-hour glucose gene variants and physical activity or BMI on 2-hour glucose levels. Diabetes.

[31] Wu P, Rybin D, Bielak LF, Feitosa MF, Franceschini N, Li Y (2020). Smoking-by-genotype interaction in type 2 diabetes risk and fasting glucose. PLoS ONE.

[32] Cauchi S, Choquet H, Gutiérrez-Aguilar R, Capel F, Grau K, Proença C (2008). Effects of TCF7L2 polymorphisms on obesity in European populations. Obesity (Silver Spring).

[33] Florez JC, Jablonski KA, Bayley N, Pollin TI, de Bakker PI, Shuldiner AR (2006). TCF7L2 polymorphisms and progression to diabetes in the Diabetes Prevention Program. N Engl J Med.

[34] McCaffery JM, Jablonski KA, Franks PW, Dagogo-Jack S, Wing RR, Knowler WC (2011). TCF7L2 polymorphism, weight loss and proinsulin:insulin ratio in the diabetes prevention program. PLoS ONE.

[35] Page MJ, McKenzie JE, Bossuyt PM, Boutron I, Hoffmann TC, Mulrow CD (2021). The PRISMA 2020 statement: an updated guideline for reporting systematic reviews. BMJ.

[36] Sterne JAC, Savović J, Page MJ, Elbers RG, Blencowe NS, Boutron I (2019). RoB 2: a revised tool for assessing risk of bias in randomised trials. BMJ.

[37] Wells G SB, O'Connell D, et al. the Newcastle-Ottawa Scale (NOS) for assessing the quality of nonrandomised in meta-analyses. Ottawa, ON: Ottawa hospital Institute; 2011.

[38] Gjesing AP, Kjems LL, Vestmar MA, Grarup N, Linneberg A, Deacon CF (2011). Carriers of the TCF7L2 rs7903146 TT genotype have elevated levels of plasma glucose, serum proinsulin and plasma gastric inhibitory polypeptide (GIP) during a meal test. Diabetologia.

[39] Daniele G, Gaggini M, Comassi M, Bianchi C, Basta G, Dardano A (2015). Glucose Metabolism in High-Risk Subjects for Type 2 Diabetes Carrying the rs7903146 TCF7L2 Gene Variant. J Clin Endocrinol Metab.

[40] Ferreira MC, da Silva MER, Fukui RT, Arruda-Marques MDC, Dos Santos RF (2018). TCF7L2 correlation in both insulin secretion and postprandial insulin sensitivity. Diabetol Metab Syndr.

[41] Pilgaard K, Jensen CB, Schou JH, Lyssenko V, Wegner L, Brøns C (2009). The T allele of rs7903146 TCF7L2 is associated with impaired insulinotropic action of incretin hormones, reduced 24 h profiles of plasma insulin and glucagon, and increased hepatic glucose production in young healthy men. Diabetologia.

[42] Adamska E, Kretowski A, Goscik J, Citko A, Bauer W, Waszczeniuk M (2018). The type 2 diabetes susceptibility TCF7L2 gene variants affect postprandial glucose and fat utilization in non-diabetic subjects. Diabetes Metab.

[43] López-Ortiz MM, Garay-Sevilla ME, Tejero ME, Perez-Luque EL (2016). Analysis of the interaction between transcription factor 7-like 2 genetic variants with nopal and wholegrain fibre intake: effects on anthropometric and metabolic characteristics in type 2 diabetes patients. Br J Nutr.

[44] Guevara-Cruz M, Tovar AR, Aguilar-Salinas CA, Medina-Vera I, Gil-Zenteno L, Hernández-Viveros I (2012). A dietary pattern including nopal, chia seed, soy protein, and oat reduces serum triglycerides and glucose intolerance in patients with metabolic syndrome. J Nutr.

[45] Rezazadeh K, Rahmati-Yamchi M, Mohammadnejad L, Ebrahimi-Mameghani M (2018). Effects of artichoke leaf extract supplementation on metabolic parameters in women with metabolic syndrome: Influence of TCF7L2-rs7903146 and FTO-rs9939609 polymorphisms. Phytother Res.

[46] Bo S, Gambino R, Ciccone G, Rosato R, Milanesio N, Villois P (2009). Effects of TCF7L2 polymorphisms on glucose values after a lifestyle intervention. Am J Clin Nutr.

[47] Haupt A, Thamer C, Heni M, Ketterer C, Machann J, Schick F (2010). Gene variants of TCF7L2 influence weight loss and body composition during lifestyle intervention in a population at risk for type 2 diabetes. Diabetes.

[48] Walker CG, Loos RJ, Mander AP, Jebb SA, Frost GS, Griffin BA (2012). Genetic predisposition to type 2 diabetes is associated with impaired insulin secretion but does not modify insulin resistance or secretion in response to an intervention to lower dietary saturated fat. Genes Nutr.

[49] Ebrahimi-Mameghani M, Asghari-Jafarabadi M, Rezazadeh K (2018). TCF7L2-rs7903146 polymorphism modulates the effect of artichoke leaf extract supplementation on insulin resistance in metabolic syndrome: a randomized, double-blind, placebo-controlled trial. J Integr Med.

[50] Ruchat SM, Rankinen T, Weisnagel SJ, Rice T, Rao DC, Bergman RN (2010). Improvements in glucose homeostasis in response to regular exercise are influenced by the PPARG Pro12Ala variant: results from the HERITAGE Family Study. Diabetologia.

[51] Ruchat SM, Elks CE, Loos RJ, Vohl MC, Weisnagel SJ, Rankinen T (2009). Evidence of interaction between type 2 diabetes susceptibility genes and dietary fat intake for adiposity and glucose homeostasis-related phenotypes. J Nutrigenet Nutrigenomics.

[52] Nettleton JA, McKeown NM, Kanoni S, Lemaitre RN, Hivert MF, Ngwa J (2010). Interactions of dietary whole-grain intake with fasting glucose- and insulin-related genetic loci in individuals of European descent: a meta-analysis of 14 cohort studies. Diabetes Care.

[53] Delgado-Lista J, Perez-Martinez P, García-Rios A, Phillips CM, Williams CM, Gulseth HL (2011). Pleiotropic effects of TCF7L2 gene variants and its modulation in the metabolic syndrome: From the LIPGENE study. Atherosclerosis.

[54] Hindy G, Sonestedt E, Ericson U, Jing XJ, Zhou Y, Hansson O (2012). Role of TCF7L2 risk variant and dietary fibre intake on incident type 2 diabetes. Diabetologia.

[55] Corella D, Carrasco P, Sorlí JV, Estruch R, Rico-Sanz J, Martínez-González M (2013). Mediterranean diet reduces the adverse effect of the TCF7L2-rs7903146 polymorphism on cardiovascular risk factors and stroke incidence: a randomized controlled trial in a high-cardiovascular-risk population. Diabetes Care.

[56] Ouhaibi-Djellouli H, Mediene-Benchekor S, Lardjam-Hetraf SA, Hamani-Medjaoui I, Meroufel DN, Boulenouar H (2014). The TCF7L2 rs7903146 polymorphism, dietary intakes and type 2 diabetes risk in an Algerian population. BMC genet.

[57] Lu J, Varghese RT, Zhou L, Vella A, Jensen MD (2017). Glucose tolerance and free fatty acid metabolism in adults with variations in TCF7L2 rs7903146. Metabolism.

[58] Bodhini D, Gaal S, Shatwan I, Ramya K, Ellahi B, Surendran S (2017). Interaction between TCF7L2 polymorphism and dietary fat intake on high density lipoprotein cholesterol. PloS One.

[59] Barabash A, Valerio JD, Garcia de la Torre N, Jimenez I, Del Valle L, Melero V, et al. TCF7L2 rs7903146 polymorphism modulates the association between adherence to a Mediterranean diet and the risk of gestational diabetes mellitus. Metabol Open. 2020;8:100069. 10.1016/j.metop.2020.100069 PMC7718167 33305252

[60] Bauer W, Adamska-Patruno E, Krasowska U, Moroz M, Fiedorczuk J, Czajkowski P (2021). Dietary Macronutrient Intake May Influence the Effects of TCF7L2 rs7901695 Genetic Variants on Glucose Homeostasis and Obesity-Related Parameters: A Cross-Sectional Population-Based Study. Nutrients.

[61] Phillips CM, Goumidi L, Bertrais S, Field MR, McManus R, Hercberg S (2012). Dietary saturated fat, gender and genetic variation at the TCF7L2 locus predict the development of metabolic syndrome. J Nutr Biochem.

[62] Brito EC, Lyssenko V, Renström F, Berglund G, Nilsson PM, Groop L (2009). Previously associated type 2 diabetes variants may interact with physical activity to modify the risk of impaired glucose regulation and type 2 diabetes: a study of 16,003 Swedish adults. Diabetes.

[63] Lin WY, Liu YL, Yang AC, Tsai SJ, Kuo PH (2020). Active Cigarette Smoking Is Associated With an Exacerbation of Genetic Susceptibility to Diabetes. Diabetes.

[64] Jung SY, Sobel EM, Papp JC, Crandall CJ, Fu AN, Zhang ZF (2016). Obesity and associated lifestyles modify the effect of glucose metabolism-related genetic variants on impaired glucose homeostasis among postmenopausal women. Genet Epidemiol.

[65] Dujic T, Bego T, Malenica M, Velija-Asimi Z, Ahlqvist E, Groop L (2019). Effects of TCF7L2 rs7903146 variant on metformin response in patients with type 2 diabetes. Bosn J Basic Med Sci.

[66] Roncero-Ramos I, Gutierrez-Mariscal FM, Gomez-Delgado F, Villasanta-Gonzalez A, Torres-Peña JD, Cruz-Ares S (2021). Beta cell functionality and hepatic insulin resistance are major contributors to type 2 diabetes remission and starting pharmacological therapy: from CORDIOPREV randomized controlled trial. Transl Res.

[67] Kumar A, Sundaram K, Mu J, Dryden GW, Sriwastva MK, Lei C (2021). High-fat diet-induced upregulation of exosomal phosphatidylcholine contributes to insulin resistance. Nat Commun.

[68] Hernández E, Kahl S, Seelig A, Begovatz P, Irmler M, Kupriyanova Y (2017). Acute dietary fat intake initiates alterations in energy metabolism and insulin resistance. J Clin Inves.

[69] Martinez JA, Navas-Carretero S, Saris WH, Astrup A (2014). Personalized weight loss strategies-the role of macronutrient distribution. Nat Rev Endocrinol.

