# Supplementary material for: Effect of TCF7L2 on the relationship between lifestyle factors and glycemic parameters: a systematic review
Source: Nutr J. 2022 Sep 26;21:59. doi: 10.1186/s12937-022-00813-w (PMC9511734; doi:10.1186/s12937-022-00813-w)
Supplement: Supplementary file 1 — Additional file 1: Table S1. Details of the search strategy in electronic databases. Table S2. Quality assessment of studies based on gene-lifestyle interaction on glycaemic parameters. Table S3. Quality assessment of cohort studies by using the Rob2 tool. Table S4. Quality assessment of cross-sectional studies by using the Newcastle Ottawa Scale. Table S5. Quality assessment of cohort studies by using the Newcastle Ottawa Scale [file 12937_2022_813_MOESM1_ESM.docx]

| **Table S1.** Details of the search strategy in electronic databases | |
| --- | --- |
| PubMed | (TCF7L2 OR “Transcription Factor 7 Like 2 Protein” OR “T-Cell-Specific Transcription Factor 4” OR “T Cell Specific Transcription Factor 4” OR “T Cell Transcription Factor 4” OR “TCF7L2 Transcription Factor” OR (Transcription Factor AND TCF7L2) OR “T Cell Factor 4” OR tcf7l2)  AND  (insulin OR “Insulin Resistance” OR (Resistance AND Insulin) OR “Insulin Sensitivity” OR (Sensitivity AND Insulin) OR “Glucose Intolerance” OR “Glucose Intolerances” OR (Intolerance AND Glucose) OR (Intolerances AND Glucose) OR “Impaired Glucose Tolerance” OR (Glucose Tolerance AND Impaired) OR (Glucose Tolerances AND Impaired) OR “Impaired Glucose Tolerances” OR (Tolerance AND Impaired Glucose) OR (Tolerances AND Impaired Glucose)  OR “Glucose Tolerance Test” OR “Oral Glucose Tolerance Test” OR OGTT OR “Oral Glucose Tolerance” OR (Glucose Tolerance AND Oral) OR “Intravenous Glucose Tolerance Test” OR “Intravenous Glucose Tolerance” OR HOMA OR HOMA-IR OR Insulinemia OR “Glucose Metabolism Disorders” OR (Disorder AND Glucose Metabolism) OR (Disorders AND Glucose Metabolism) OR (Metabolism Disorder AND Glucose) OR (Metabolism Disorders AND Glucose) OR “Glucose Metabolic Disorders” OR (Disorder AND Glucose Metabolic) OR (Disorders AND Glucose Metabolic) OR (Metabolic Disorder AND Glucose) OR (Metabolic Disorders AND Glucose) OR “Glucose Metabolism Disorder” OR “Glucose Metabolic Disorder” OR “Hyperglycemia” OR “Hyperglycemias” OR (Hyperglycemia AND Postprandial) OR (Hyperglycemias AND Postprandial) OR “Postprandial Hyperglycemias” OR “Postprandial Hyperglycemia” OR “Blood Glucose” OR “Blood Sugar” OR (Sugar AND Blood) OR (Glucose AND Blood) OR “Glycated Hemoglobin A” OR (Hemoglobin A AND Glycated) OR “Hb A1c” OR “Glycosylated Hemoglobin A” OR (Hemoglobin A AND Glycosylated) OR “Glycohemoglobin A” OR “Hemoglobin A” OR “Hemoglobin AND Glycated A1” OR “Hemoglobin AND Glycated” OR “Glycated A1a-2 Hemoglobin” OR (Hemoglobin AND Glycated A1a 2) OR (Hemoglobin AND Glycosylated A1) OR (Hemoglobin AND Glycosylated) OR “Glycosylated A1a-1 Hemoglobin” OR (Hemoglobin AND Glycosylated) OR (Hemoglobin AND Glycated A1b) OR (A1b Hemoglobin AND Glycated) OR “Glycated A1b Hemoglobin” OR “Hb A1b” OR (Hemoglobin AND Glycosylated A1b) OR (A1b Hemoglobin AND Glycosylated) OR “Glycosylated A1b Hemoglobin” OR “Glycated Hemoglobin A1c” OR (Hemoglobin A1c AND Glycated) OR “Glycosylated Hemoglobin A1c” OR (Hemoglobin A1c AND Glycosylated) OR “Glycated Hemoglobins” OR (Hemoglobins AND Glycated) OR (Hemoglobin AND Glycosylated) OR “Glycosylated Hemoglobin” OR “glucosylated hemoglobin A” OR (hemoglobin A AND glucosylated) OR “glucosylated serum albumin” OR “N-acetylglucosamine-BSA”)  AND  (Macronutrient OR micronutrient OR intake OR consumption OR vitamin OR mineral OR sugar OR protein OR carbohydrate OR fat* OR lipid OR "fatty acid" OR "saturated fat*" OR SFA OR MUFA OR PUFA OR (Acids AND Trans Fatty) OR (Fatty Acids AND Trans) OR "Trans-Fatty Acids" OR (Acids AND Trans-Fatty) OR (Acids AND Unsaturated Fatty) OR "unsaturated Fatty Acids" OR "Polyunsaturated Fatty Acids" OR (Acids AND Polyunsaturated Fatty) OR (Fatty Acids AND Polyunsaturated) OR fibre OR fiber OR "Dietary Fibers" OR (Fibers AND Dietary) OR (Fiber AND Dietary) OR "Wheat Bran" OR (Bran AND Wheat) OR (Brans AND Wheat) OR "Wheat Brans" OR "Roughage" OR "Roughages" OR grain* OR "glycaemic index" OR "glycaemic load" OR "glycemic index" OR "glycemic load" OR magnesium OR zinc OR iron OR vitamin OR "refined grain" OR "whole grain" OR bread OR rice OR vegetable OR cereal OR pasta OR coffee OR potato OR fruit OR nuts OR legume OR bean OR egg OR beverage OR "sugar-sweetened beverages" OR dairy OR dairies OR milk OR cheese OR yogurt OR butter OR meat OR fish OR seafood OR "processed meat" OR "fast food" OR "dietary pattern" OR "dietary habit" OR "dietary change" OR "Mediterranean score" OR "Mediterranean diet" OR "traditional pattern" OR "prudent pattern" OR "western pattern" OR diet* OR nutrition* OR nutrient OR energy OR caloric OR calorie OR fat OR meal OR dietary OR nutrition OR nutritional OR "bed rest" OR (Rest AND Bed) OR (Rests AND Bed) OR Bedrest OR "Life Styles" OR Lifestyle OR Lifestyles OR exercise* OR "physical activity" OR "energy expenditure" OR sport OR "metabolic equivalent" OR smoke*) |
| Scopus | (ALL(“Transcription Factor 7 Like 2 Protein”) OR ALL(“T-Cell-Specific Transcription Factor 4”) OR ALL(“T Cell Specific Transcription Factor 4”) OR ALL(“T Cell Transcription Factor 4”) OR ALL(“TCF7L2 Transcription Factor”) OR (ALL(Transcription Factor) AND ALL(TCF7L2)) OR ALL(“T Cell Factor 4”) OR ALL(tcf7l2))  AND  (ALL(insulin) OR ALL(“Insulin Resistance”) OR (ALL(Resistance) AND ALL(Insulin)) OR ALL(“Insulin Sensitivity”) OR (ALL(Sensitivity) AND ALL(Insulin)) OR ALL(“Glucose Intolerance”) OR ALL(“Glucose Intolerances”) OR (ALL(Intolerance) AND ALL(Glucose)) OR (ALL(Intolerances) AND ALL(Glucose)) OR ALL(“Impaired Glucose Tolerance”) OR (ALL(Glucose Tolerance) AND ALL(Impaired)) OR (ALL(Glucose Tolerances) AND ALL(Impaired)) OR ALL(“Impaired Glucose Tolerances”) OR (ALL(Tolerance) AND ALL(Impaired Glucose)) OR (ALL(Tolerances) AND ALL(Impaired Glucose)) OR ALL(“Glucose Tolerance Test”) OR ALL(“Oral Glucose Tolerance Test”) OR ALL(OGTT) OR ALL(“Oral Glucose Tolerance”) OR (ALL(Glucose Tolerance) AND ALL(Oral)) OR ALL(“Intravenous Glucose Tolerance Test”) OR ALL(“Intravenous Glucose Tolerance”) OR ALL(HOMA) OR ALL(HOMA-IR) OR ALL(Insulinemia) OR ALL(“Glucose Metabolism Disorders”) OR (ALL(Disorder) AND ALL(Glucose Metabolism)) OR (ALL(Disorders) AND ALL(Glucose Metabolism)) OR (ALL(Metabolism Disorder) AND ALL(Glucose)) OR (ALL(Metabolism Disorders) AND ALL(Glucose)) OR ALL(“Glucose Metabolic Disorders”) OR (ALL(Disorder) AND ALL(Glucose Metabolic)) OR (ALL(Disorders) AND ALL(Glucose Metabolic)) OR (ALL(Metabolic Disorder) AND ALL(Glucose)) OR (ALL(Metabolic Disorders) AND ALL(Glucose)) OR ALL(“Glucose Metabolism Disorder”) OR ALL(“Glucose Metabolic Disorder”) OR ALL(Hyperglycemia) OR ALL(Hyperglycemias) OR (ALL(Hyperglycemia) AND ALL(Postprandial)) OR (ALL(Hyperglycemias) AND ALL(Postprandial)) OR ALL(“Postprandial Hyperglycemias”) OR ALL(“Postprandial Hyperglycemia”) OR ALL(“Blood Glucose”) OR ALL(“Blood Sugar”) OR (ALL(Sugar) AND ALL(Blood)) OR (ALL(Glucose) AND ALL(Blood)) OR ALL(“Glycated Hemoglobin A”) OR (ALL(“Hemoglobin A”) AND ALL(Glycated)) OR ALL(“Hb A1c”) OR ALL(“Glycosylated Hemoglobin A”) OR (ALL(“Hemoglobin A”) AND ALL(Glycosylated)) OR ALL(“Glycohemoglobin A”) OR ALL(“Hemoglobin A”) OR (ALL(Hemoglobin) AND ALL(Glycated A1)) OR (ALL(Hemoglobin) AND ALL(Glycated)) OR ALL(“Glycated A1a-2 Hemoglobin”) OR (ALL(Hemoglobin) AND ALL(Glycated A1a 2)) OR (ALL(Hemoglobin) AND ALL(Glycosylated A1)) OR (ALL(Hemoglobin) AND ALL(Glycosylated)) OR ALL(“Glycosylated A1a-1 Hemoglobin”) OR (ALL(Hemoglobin) AND ALL(Glycosylated)) OR (ALL(Hemoglobin) AND ALL(Glycated A1b)) OR (ALL(“A1b Hemoglobin”) AND ALL(Glycated)) OR ALL(“Glycated A1b Hemoglobin”) OR ALL(“Hb A1b”) OR (ALL(Hemoglobin) AND ALL(Glycosylated A1b)) OR (ALL(A1b Hemoglobin) AND ALL(Glycosylated)) OR ALL(“Glycosylated A1b Hemoglobin”) OR ALL(“Glycated Hemoglobin A1c”)  OR (ALL(Hemoglobin A1c) AND ALL(Glycated)) OR ALL(“Glycosylated Hemoglobin A1c”) OR (ALL(Hemoglobin A1c) AND ALL(Glycosylated)) OR ALL(“Glycated Hemoglobins”) OR (ALL(Hemoglobins) AND ALL(Glycated)) OR (ALL(Hemoglobin) AND ALL(Glycosylated)) OR ALL(“Glycosylated Hemoglobin”) OR ALL(“glucosylated hemoglobin A”) OR (ALL(hemoglobin A) AND ALL(glucosylated)) OR ALL(“glucosylated serum albumin”) OR ALL(“N-acetylglucosamine-BSA”))  AND  (ALL(Macronutrient) OR ALL(micronutrient) OR ALL(intake) OR ALL(consumption) OR ALL(vitamin) OR ALL(mineral) OR ALL(sugar) OR ALL(protein) OR ALL(carbohydrate) OR ALL(fat*) OR ALL(lipid) OR ALL(“fatty acid”) OR ALL(“saturated fat*”) OR ALL(SFA) OR ALL(MUFA) OR ALL(PUFA) OR (ALL(Acids) AND ALL(Trans Fatty)) OR (ALL(Fatty Acids) AND ALL(Trans)) OR ALL(“Trans-Fatty Acids”) OR (ALL(Acids) AND ALL(Trans-Fatty)) OR (ALL(Acids) AND ALL(Unsaturated Fatty)) OR ALL(“Unsaturated Fatty Acids”) OR ALL(“Polyunsaturated Fatty Acids”) OR (ALL(Acids) AND ALL(Polyunsaturated Fatty)) OR (ALL(Fatty Acids) AND ALL(Polyunsaturated)) OR ALL(fibre) OR ALL(fiber) OR ALL(“Dietary Fibers”) OR (ALL(Fibers) AND ALL(Dietary)) OR (ALL(Fiber) AND ALL(Dietary)) OR ALL(“Wheat Bran”) OR (ALL(Bran) AND ALL(Wheat)) OR (ALL(Brans) AND ALL(Wheat)) OR ALL(“Wheat Brans”) OR ALL(Roughage) OR ALL(Roughages) OR ALL(grain*) OR ALL(“glycaemic index”) OR ALL(“glycaemic load”) OR ALL(“glycemic index”) OR ALL(“glycemic load”) OR ALL(magnesium) OR ALL(zinc) OR ALL(iron) OR ALL(“refined grain”) OR ALL(“whole grain”) OR ALL(bread) OR ALL(rice) OR ALL(vegetable) OR ALL(cereal) OR ALL(pasta) OR ALL(coffee) OR ALL(potato*) OR ALL(fruit) OR ALL(nuts) OR ALL(legume) OR ALL(bean) OR ALL(egg) OR ALL(beverage) OR ALL(“sugar-sweetened beverages”) OR ALL(dairy) OR ALL(dairies) OR ALL(milk) OR ALL(cheese) OR ALL(yogurt) OR ALL(butter) OR ALL(meat) OR ALL(fish) OR ALL(seafood) OR ALL(“processed meat”) OR ALL(“fast food”) OR ALL(“dietary pattern”) OR ALL(“dietary habit”) OR ALL(“dietary change”) OR ALL(“Mediterranean score”) OR ALL(“Mediterranean diet”) OR ALL(“traditional pattern”) OR ALL(“prudent pattern”) OR ALL(“western pattern”) OR ALL(diet*) OR ALL(nutrition*) OR ALL(nutrient) OR ALL(energy) OR ALL(caloric) OR ALL(calorie) OR ALL(fat) OR ALL(meal) OR ALL(dietary) OR ALL(nutrition) OR ALL(nutritional) OR ALL(“bed rest”) OR (ALL(Rest) AND ALL(Bed)) OR (ALL(Rests) AND ALL(Bed)) OR ALL(Bedrest) OR ALL(“Life Styles”) OR ALL(Lifestyle) OR ALL(Lifestyles) OR ALL(exercise*) OR ALL(“physical activity”) OR ALL(“energy expenditure”) OR ALL(sport) OR ALL(“metabolic equivalent”) OR ALL(smoke*)) |
| Web of Science | (ALL=(“T Cell Specific Transcription Factor 4”) OR ALL=(“T Cell Transcription Factor 4”) OR ALL=(“T Cell Factor 4”) OR ALL=(tcf7l2))  AND  (ALL=(insulin) OR ALL=(“Glucose Intolerance”) OR (AB=(Intolerances) AND ALL=(Glucose)) OR (ALL=(Tolerance) AND ALL=(Impaired Glucose)) OR ALL=(“Glucose Tolerance Test”) OR ALL=(OGTT) OR (ALL=(Glucose Tolerance) AND ALL=(Oral)) OR ALL=(“Intravenous Glucose Tolerance”) OR ALL=(HOMA) OR ALL=(Insulinemia) OR (ALL=(Metabolism Disorder) AND ALL=(Glucose)) OR (ALL=(Metabolism Disorders) AND ALL=(Glucose)) OR (ALL=(Metabolic Disorder) AND ALL=(Glucose)) OR (AB=(Metabolic Disorders) AND ALL=(Glucose)) OR ALL=(Hyperglycemia) OR ALL=(Hyperglycemias) OR (ALL=(Sugar) AND ALL=(Blood)) OR (AB=(Glucose) AND ALL=(Blood)) OR ALL=(“Hb A1c”) OR ALL=(“Glycohemoglobin A”) OR ALL=(“Hemoglobin A”) OR (ALL=(Hemoglobin) AND ALL=(Glycated)) OR (ALL=(Hemoglobin) AND ALL=(Glycosylated)) OR (ALL=(hemoglobin A) AND ALL=(glucosylated)) OR ALL=(“glucosylated serum albumin”) OR ALL=(“N-acetylglucosamine-BSA”))  AND  (ALL=(macronutrient) OR ALL=(micronutrient) OR ALL=(sugar) OR ALL=(carbohydrate) OR ALL=(fat) OR All=(protein) OR ALL=(lipid) OR ALL=(“fatty acid”) OR ALL=(SFA) OR ALL=(MUFA) OR ALL=(PUFA) OR ALL=(“refined grain”) OR ALL=(“whole grain”) OR ALL=(bread) OR ALL=(rice) OR ALL=(vegetable) OR ALL=(cereal) OR ALL=(pasta) OR ALL=(potato) OR ALL=(fruit) OR ALL=(nut) OR ALL=(legume) OR ALL=(bean) OR ALL=(egg) OR ALL=(beverage) OR ALL=(dairy) OR ALL=(dairies) OR ALL=(milk) OR ALL=(cheese) OR ALL=(yogurt) OR ALL=(butter) OR ALL=(meat) OR ALL=(fish) OR ALL=(seafood) OR ALL=(“fast food”) OR ALL=(diet*) OR ALL=(nutrition*) OR ALL=(energy) OR ALL=(caloric) OR ALL=(calorie) OR ALL=(meal) OR ALL=(nutrition) OR ALL=(“bed rest”) OR (ALL=(Rest) AND ALL=(Bed)) OR (ALL=(Rests) AND ALL=(Bed)) OR ALL=(“Life Styles”) OR ALL=(Lifestyle) OR ALL=(Lifestyles) OR ALL=(exercise) OR ALL=(“physical activity”) OR ALL=(smoke*)) |
| Embase | tcf7l2  AND  (insulin OR (Insulin Resistance) OR (Resistance AND Insulin) OR (Insulin Sensitivity) OR (Sensitivity AND Insulin) OR (Glucose Intolerance) OR (Glucose Intolerances) OR (Intolerance AND Glucose) OR (Intolerances AND Glucose) OR (Impaired Glucose Tolerance) OR (Glucose Tolerance AND Impaired) OR (Glucose Tolerances AND Impaired) OR (Impaired Glucose Tolerances) OR (Tolerance AND Impaired Glucose) OR (Tolerances AND Impaired Glucose)  OR (Glucose Tolerance Test) OR (Oral Glucose Tolerance Test) OR OGTT OR (Oral Glucose Tolerance) OR (Glucose Tolerance AND Oral) OR (Intravenous Glucose Tolerance Test) OR (Intravenous Glucose Tolerance) OR HOMA OR HOMA-IR OR Insulinemia OR (Glucose Metabolism Disorders) OR (Disorder AND Glucose Metabolism) OR (Disorders AND Glucose Metabolism) OR (Metabolism Disorder AND Glucose) OR (Metabolism Disorders AND Glucose) OR (Glucose Metabolic Disorders) OR (Disorder AND Glucose Metabolic) OR (Disorders AND Glucose Metabolic) OR (Metabolic Disorder AND Glucose) OR (Metabolic Disorders AND Glucose) OR (Glucose Metabolism Disorder) OR (Glucose Metabolic Disorder) OR Hyperglycemia OR Hyperglycemias OR (Hyperglycemia AND Postprandial) OR (Hyperglycemias AND Postprandial) OR (Postprandial Hyperglycemias) OR (Postprandial Hyperglycemia) OR (Blood Glucose) OR (Blood Sugar) OR (Sugar AND Blood) OR (Glucose AND Blood) OR (Glycated Hemoglobin A) OR (Hemoglobin A AND Glycated) OR (Hb A1c) OR (Glycosylated Hemoglobin A) OR (Hemoglobin A AND Glycosylated) OR (Glycohemoglobin A) OR (Hemoglobin A) OR (Hemoglobin AND Glycated A1) OR (Hemoglobin AND Glycated) OR (Glycated A1a-2 Hemoglobin) OR (Hemoglobin AND Glycated A1a 2) OR (Hemoglobin AND Glycosylated A1) OR (Hemoglobin AND Glycosylated) OR (Glycosylated A1a-1 Hemoglobin) OR (Hemoglobin AND Glycosylated) OR (Hemoglobin AND Glycated A1b) OR (A1b Hemoglobin AND Glycated) OR (Glycated A1b Hemoglobin) OR (Hb A1b) OR (Hemoglobin AND Glycosylated A1b) OR (A1b Hemoglobin AND Glycosylated) OR (Glycosylated A1b Hemoglobin) OR (Glycated Hemoglobin A1c) OR (Hemoglobin A1c AND Glycated) OR (Glycosylated Hemoglobin A1c) OR (Hemoglobin A1c AND Glycosylated) OR (Glycated Hemoglobins) OR (Hemoglobins AND Glycated) OR (Hemoglobin AND Glycosylated) OR (Glycosylated Hemoglobin) OR (glucosylated hemoglobin A) OR (hemoglobin A AND glucosylated) OR (glucosylated serum albumin) OR (N-acetylglucosamine-BSA))  AND  (Macronutrient OR micronutrient OR intake OR consumption OR vitamin OR mineral OR sugar OR protein OR carbohydrate OR fat* OR lipid OR "fatty acid" OR "saturated fat*" OR SFA OR MUFA OR PUFA OR (Acids AND Trans Fatty) OR (Fatty Acids AND Trans) OR "Trans-Fatty Acids" OR (Acids AND Trans-Fatty) OR (Acids AND Unsaturated Fatty) OR "unsaturated Fatty Acids" OR "Polyunsaturated Fatty Acids" OR (Acids AND Polyunsaturated Fatty) OR (Fatty Acids AND Polyunsaturated) OR fibre OR fiber OR "Dietary Fibers" OR (Fibers AND Dietary) OR (Fiber AND Dietary) OR "Wheat Bran" OR (Bran AND Wheat) OR (Brans AND Wheat) OR "Wheat Brans" OR "Roughage" OR "Roughages" OR grain* OR "glycaemic index" OR "glycaemic load" OR "glycemic index" OR "glycemic load" OR magnesium OR zinc OR iron OR vitamin OR "refined grain" OR "whole grain" OR bread OR rice OR vegetable OR cereal OR pasta OR coffee OR potato OR fruit OR nuts OR legume OR bean OR egg OR beverage OR "sugar-sweetened beverages" OR dairy OR dairies OR milk OR cheese OR yogurt OR butter OR meat OR fish OR seafood OR "processed meat" OR "fast food" OR "dietary pattern" OR "dietary habit" OR "dietary change" OR "Mediterranean score" OR "Mediterranean diet" OR "traditional pattern" OR "prudent pattern" OR "western pattern" OR diet* OR nutrition* OR nutrient OR energy OR caloric OR calorie OR fat OR meal OR dietary OR nutrition OR nutritional OR "bed rest" OR (Rest AND Bed) OR (Rests AND Bed) OR Bedrest OR "Life Styles" OR Lifestyle OR Lifestyles OR exercise* OR "physical activity" OR "energy expenditure" OR sport OR "metabolic equivalent" OR smoke*) |

| Table S2: Quality assessment of studies based on gene-lifestyle interaction on glycaemic parameters | | | | | | | | | |  |
| --- | --- | --- | --- | --- | --- | --- | --- | --- | --- | --- |
| study | Interaction as primary study goal | Statistical test for interaction | Correction for multiple testing | Correction for ethnicity | Hardy-Weinberg Equilibrium | Test of group similarity at baseline | Sample size | Sufficient study  details | score | overall quality |
| Adamska et al, 2017 | 1 | 0 | 1 | 1 | 1 | 1 | -1 | 1 | 5 | Intermediate |
| Alibegovic et al, 2010 | 1 | 0 | 0 | 1 | -1 | 1 | -1 | 1 | 2 | Intermediate |
| Barabash et al, 2020 | 1 | 1 | 0 | 1 | 1 | 0 | -1 | 1 | 4 | Intermediate |
| Bauer et al, 2021 | 1 | 0 | 0 | 1 | 1 | 1 | -1 | 1 | 4 | Intermediate |
| Bo et al, 2009 | 1 | 1 | 1 | 1 | 1 | 1 | -1 | 1 | 6 | high |
| Bodhini et al, 2017 | 1 | 1 | 1 | 1 | 0 | 0 | 0 | 1 | 5 | high |
| Brito et al, 2009 | 1 | 1 | 1 | 1 | 1 | 0 | 1 | 1 | 7 | high |
| Cauchi et al, 2010 | 0 | 0 | 0 | -1 | 1 | 0 | -1 | 1 | 0 | Poor |
| Corella et al, 2013 | 1 | 1 | 1 | 0 | 1 | 1 | 1 | 1 | 7 | high |
| Daniele et al, 2015 | 1 | 0 | 0 | 1 | 1 | 1 | -1 | 1 | 4 | Intermediate |
| Delgado-Lista et al, 2011 | 1 | 1 | 1 | -1 | 1 | 1 | -1 | 1 | 4 | Intermediate |
| Ebrahimi-Mameghani et al, 2018 | -1 | 1 | 0 | 1 | 1 | 0 | -1 | 1 | 2 | Intermediate |
| Ferreira et al, 2018 | 1 | 0 | 1 | 1 | -1 | 1 | -1 | 1 | 3 | Intermediate |
| Florez et al, 2006 | 1 | 1 | 1 | -1 | 1 | 1 | 1 | 1 | 6 | high |
| Gjesing et al, 2011 | 1 | 1 | 1 | 1 | -1 | 1 | -1 | 1 | 4 | Intermediate |
| Grau et al, 2010 | 1 | 1 | 1 | -1 | 1 | 1 | -1 | 1 | 4 | Intermediate |
| [Guevara-Cruz](https://pubmed.ncbi.nlm.nih.gov/?sort=pubdate&term=Guevara-Cruz+M&cauthor_id=22090467) et al, 2012 | -1 | 0 | 1 | 1 | 1 | 0 | -1 | 1 | 2 | Intermediate |
| Haupt et al, 2010 | -1 | 0 | 0 | 1 | 1 | 1 | -1 | 1 | 2 | Intermediate |
| Hindy et al, 2012 | 1 | 1 | 0 | 1 | 1 | 1 | 1 | 1 | 7 | high |
| Jung et al, 2016 | 1 | 1 | 1 | 0 | 1 | -1 | 0 | 1 | 4 | Intermediate |
| Justesen et al, 2019 | 1 | 0 | 0 | 1 | 1 | -1 | -1 | 1 | 2 | Intermediate |
| Lin et al, 2020 | 1 | 1 | 0 | 1 | 1 | 0 | 1 | 1 | 6 | high |
| López-Ortiz et al, 2016 | 1 | 1 | 1 | 1 | 1 | 1 | -1 | 1 | 6 | high |
| Lu et al, 2017 | 1 | 0 | 0 | 0 | 0 | 1 | -1 | 1 | 2 | Intermediate |
| Mattei et al, 2012 | 1 | 1 | 1 | 1 | 1 | 1 | -1 | 1 | 6 | high |
| McCaffery et al, 2011 | 1 | 1 | 1 | -1 | 1 | 0 | 1 | 1 | 5 | high |
| Nettleton et al, 2010 | 1 | 1 | 1 | 0 | -1 | 0 | 1 | 1 | 4 | Intermediate |
| [Ouhaibi-Djellouli](https://pubmed.ncbi.nlm.nih.gov/?sort=date&size=200&term=Ouhaibi-Djellouli+H&cauthor_id=25491720) et al, 2014 | 1 | 1 | 1 | 1 | 1 | 1 | -1 | 1 | 6 | high |
| Perez-Martinez et al, 2012 | 1 | 1 | 0 | 0 | 1 | 1 | -1 | 1 | 4 | Intermediate |
| Phillips et al, 2012 | 1 | 1 | 0 | 0 | 1 | 1 | -1 | 1 | 4 | Intermediate |
| Pilgaard et al, 2009 | 1 | 0 | 0 | 1 | 1 | 1 | -1 | 1 | 4 | Intermediate |
| Potasso et al, 2020 | 1 | 0 | 1 | 1 | -1 | 1 | -1 | -1 | 4 | Intermediate |
| Reinehr et al, 2008 | 1 | 0 | 0 | 1 | -1 | 1 | -1 | 1 | 2 | Intermediate |
| Rezazadeh et al, 2018 | -1 | 1 | -1 | 1 | -1 | 0 | -1 | 1 | -1 | Poor |
| Ruchat et al, 2009 | 1 | 1 | 1 | 1 | 1 | -1 | -1 | 1 | 4 | Intermediate |
| Ruchat et al, 2010 | 1 | 0 | 1 | 1 | 1 | 0 | -1 | 1 | 4 | Intermediate |
| Scott et al, 2012 | 1 | 1 | 1 | 1 | -1 | 0 | 1 | 1 | 5 | Intermediate |
| Walker et al, 2012 | -1 | 1 | -1 | 1 | 1 | -1 | -1 | 1 | 0 | Poor |
| Wu et al, 2020 | 1 | 1 | 1 | 1 | -1 | 0 | 1 | 1 | 5 | Intermediate |

| Table S3: Quality assessment of cohort studies by using the Rob2 tool | | | | | | |
| --- | --- | --- | --- | --- | --- | --- |
| **First author**  **Year** | **Randomization process** | **deviations from the intended interventions** | **Missing outcome data** | **Measurement of the outcome** | **Selection of the reported result** | **Overall quality** |
| **Bo et al, 2009** | 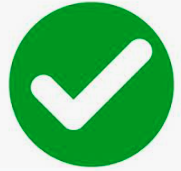 | 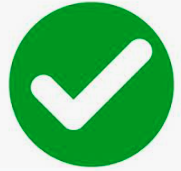 | 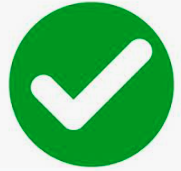 | 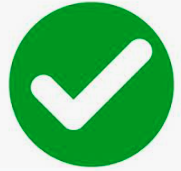 | 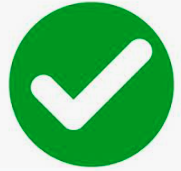 | 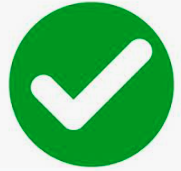 |
| **Cauchi et al, 2008** | 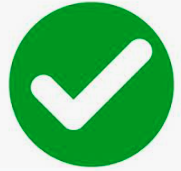 | 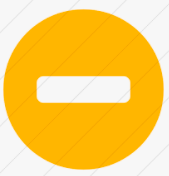 | 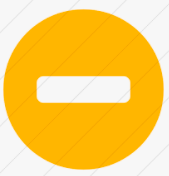 | 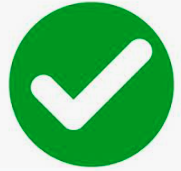 | 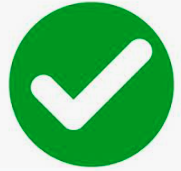 | 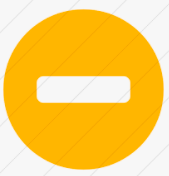 |
| **Ebrahimi-Mameghani et al, 2018** | 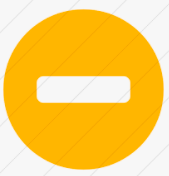 | 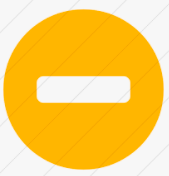 | 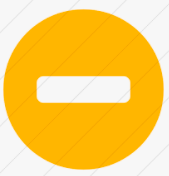 | 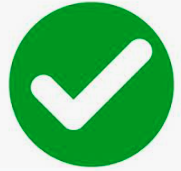 | 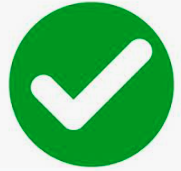 | 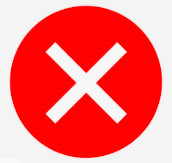 |
| **Florez et al, 2006** | 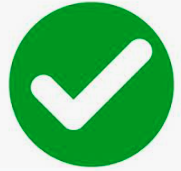 | 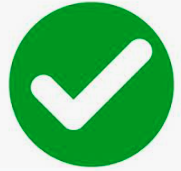 | 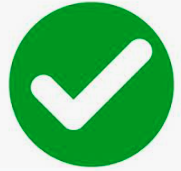 | 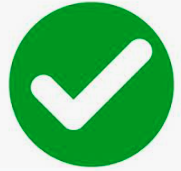 | 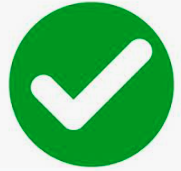 | 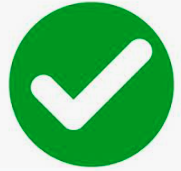 |
| **Grau et al, 2010** | 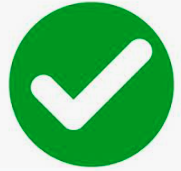 | 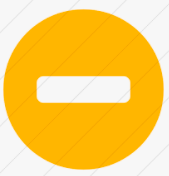 | 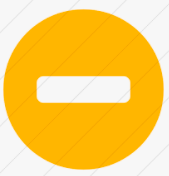 | 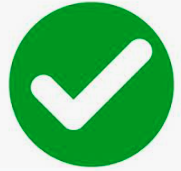 | 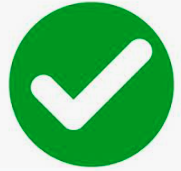 | 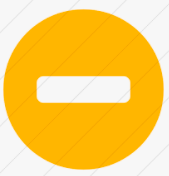 |
| **Guevara-Cruz et al, 2012** | 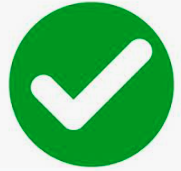 | 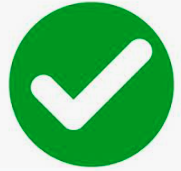 | 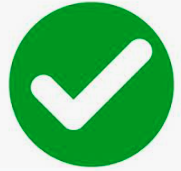 | 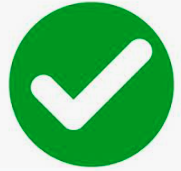 | 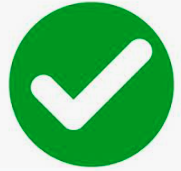 | 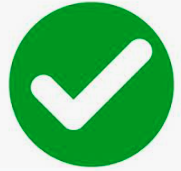 |
| **Haupt et al, 2010** | 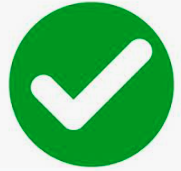 | 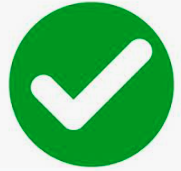 | 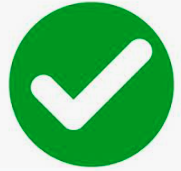 | 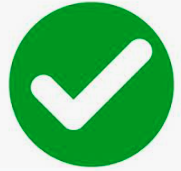 | 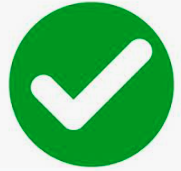 | 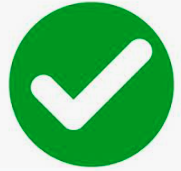 |
| **López-Ortiz et al, 2016** | 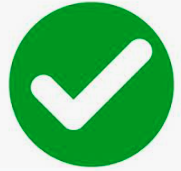 | 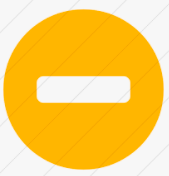 | 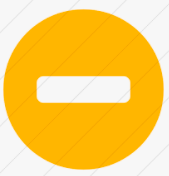 | 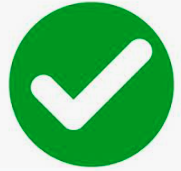 | 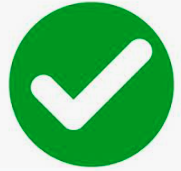 | 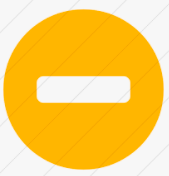 |
| **Mattei et al, 2012** | 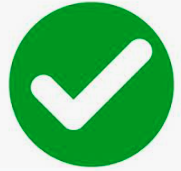 | 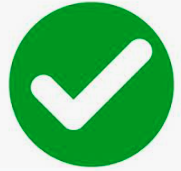 | 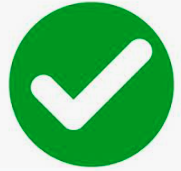 | 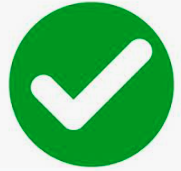 | 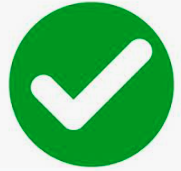 | 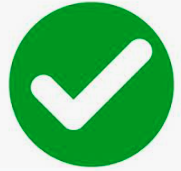 |
| **McCaffery et al, 2011** | 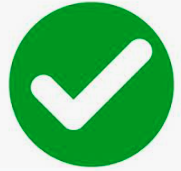 | 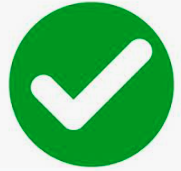 | 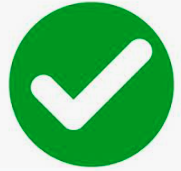 | 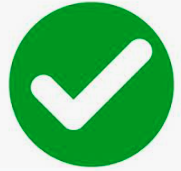 | 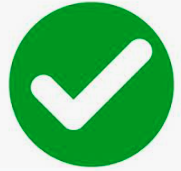 | 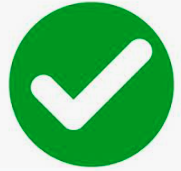 |
| **Perez-Martinez et al, 2012** | 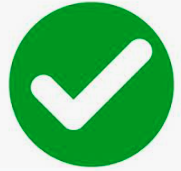 | 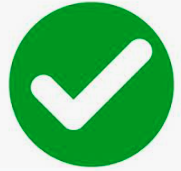 | 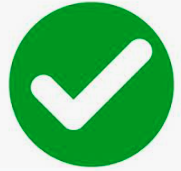 | 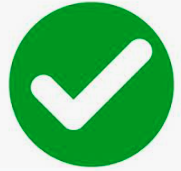 | 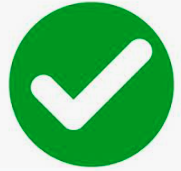 | 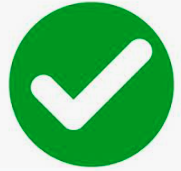 |
| **Reinehr et al, 2008** | 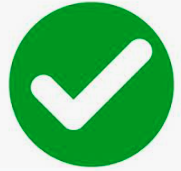 | 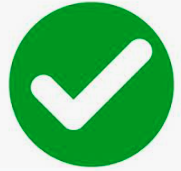 | 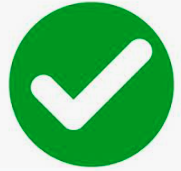 | 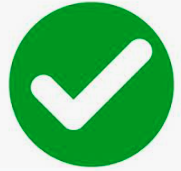 | 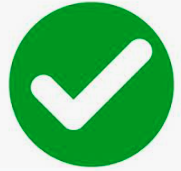 | 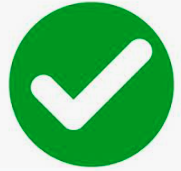 |
| **Rezazadhe et al, 2018** | 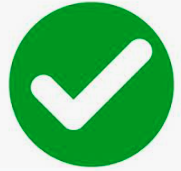 | 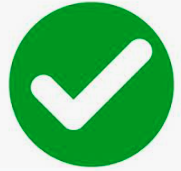 | 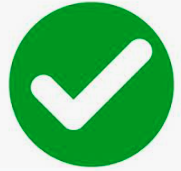 | 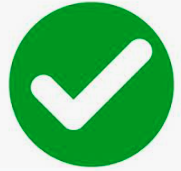 | 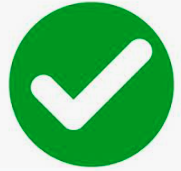 | 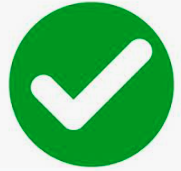 |
| **Walker et al, 2012** | 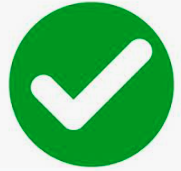 | 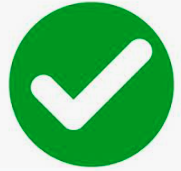 | 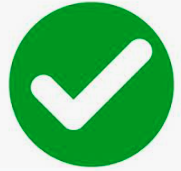 | 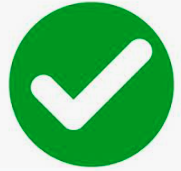 | 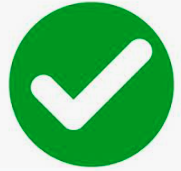 | 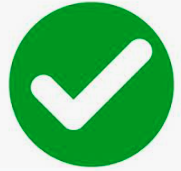 |

| Table S4: Quality assessment of cross-sectional studies by using the Newcastle Ottawa Scale | | | | | | | | |
| --- | --- | --- | --- | --- | --- | --- | --- | --- |
| Author, year (reference) | Representativeness of the sample | Sample size | Non-respondents | Ascertainment of the exposure (risk factor | Confounding factors controlled | Assessment of outcome | Statistical test | Score |
| Barabash et al, 2020 | * | * | * | ** | ** | ** | * | Very good study |
| Bauer et al, 2021 | * | * | * | - | ** | ** | * | Good study |
| Bodhing et al, 2017 | * | * | * | ** | ** | ** | * | Very good study |
| Corella et al, 2013 | * | * | * | ** | ** | * | * | Very good study |
| Delgado-Lista et al, 2011 | * | * | * | ** | -- | * | * | Good study |
| Hindy et al, 2012 | * | * | * | * | ** | ** | * | Very good study |
| Jung et al, 2016 | * | * | * | * | --- | ** | * | Good study |
| Lin et al, 2020 | * | * | * | ** | ** | ** | * | Very good study |
| Lu et al, 2017 | * |  | * | ** | * | * | * | Good study |
| Nettleton et al, 2010 | * | * | * | * | ** | ** | * | Very good study |
| [Ouhaibi-Djellouli](https://pubmed.ncbi.nlm.nih.gov/?sort=date&size=200&term=Ouhaibi-Djellouli+H&cauthor_id=25491720) et al, 2014 | * | * | * | - | ** | ** | * | Good study |
| Ruchat et al, 2009 | * | * | * | - | * | ** | * | Good quality |
| Scott et al, 2012 | * | * | * | * | * | * | * | Good quality |

| Table S5: Quality assessment of cohort studies by using the Newcastle Ottawa Scale | | | | | | | | | | |
| --- | --- | --- | --- | --- | --- | --- | --- | --- | --- | --- |
| Author, year (reference) | Representative of exposed cohort | Selection of non-exposed cohort | Valid ascertainment of exposure | Outcome of interest was not present at start of study | Control for important factors (age, sex, BMI) | Control for additional factors (energy/diet, smoking) | Assessment of outcome | Adequate duration of follow-up (≥ years) | Adequacy of follow-up | Score |
| Brito et al, 2009 | * | * | * | * | * | - | * | * | * | Good study |
| Phillips et al, 2012 | * | * | * | * | * | * | * | * | * | Good study |
| Wu et al, 2020 | * | * | * | * | * | - | * | * | * | Good study |
