# Supplementary material for: Effect of TCF7L2 on the relationship between lifestyle factors and glycemic parameters: a systematic review
Source: Nutr J. 2022 Sep 26;21:59. doi: 10.1186/s12937-022-00813-w (PMC9511734; doi:10.1186/s12937-022-00813-w)
Supplement: Supplementary file 3 — Additional file 3. [file 12937_2022_813_MOESM3_ESM.pdf]

NedMedica

EDITORIAL CERTIFICATE

This document certifies that the manuscript listed below was edited for proper English language, grammar, punctuation, spelling, and overall style by one or more of the highly qualified native English speaking editors at NedMedica

Manuscript title:

Effect of TCF7L2 on the relationship between lifestyle factors and glycemic parameters: a systematic review

Authors:

Somayeh Hosseinpour-Niazi, Parvin Mirmiran, Shabnam Hosseini, Farzad Hadaegh, Maryam S Daneshpour, Fereidoun Azizi

Date Issued:

Dec 21, 2021

CERTIFICATE Editing Certified CERTIFICATE

NedMedica®

Dr. Seyyed Mohammad Miri
Managing Director

Central Office: Kerkraderweg 13, 6416 CC, Heerlen, The Netherland Tel: +31615314198 | Fax: +31452051123
website: www.Nedmedica.nl | Email: Info@Nedmedica.nl
