# Supplementary material for: Effect of TCF7L2 on the relationship between lifestyle factors and glycemic parameters: a systematic review
Source: Nutr J. 2022 Sep 26;21:59. doi: 10.1186/s12937-022-00813-w (PMC9511734; doi:10.1186/s12937-022-00813-w)
Supplement: Supplementary file 4 — Additional file 4. [file 12937_2022_813_MOESM4_ESM.docx]

Professor Xiang Gao

Professor Qi Sun

Editors-in-chief

Nutrition Journal

**Dear Prof. Gao**

**Dear Prof. Sun**

Greetings;

Thank you very much for your letter informing us of your decision and the reviewers comments regarding the manuscript **NUTJ-D-22-00008R1,** entitled " **Effect of TCF7L2 on the relationship between lifestyle factors and glycemic parameters: a systematic review ".** The manuscript has been revised according to the referees' comments and we hope it meets your expectations. Herewith attached the revision of manuscript as well as point by point responses (R) to the reviewers’ comments(C).
